# Altered interactive dynamics of gaze behavior during face-to-face interaction in autistic individuals: a dual eye-tracking study

**DOI:** 10.1186/s13229-025-00645-5

**Published:** 2025-02-22

**Authors:** Daniel Tönsing, Bastian Schiller, Antonia Vehlen, Kathrin Nickel, Ludger Tebartz van Elst, Gregor Domes, Markus Heinrichs

**Affiliations:** 1 Department of Psychology, Laboratory for Biological Psychology, Clinical Psychology, and Psychotherapy, University of Freiburg, Freiburg, Germany; 2 Freiburg Brain Imaging Center, University Medical Center, University of Freiburg, Freiburg, Germany; 3 Department of Biological and Clinical Psychology, University of Trier, Trier, Germany; 4 Department of Psychiatry and Psychotherapy, Medical Center - University of Freiburg, Faculty of Medicine, University of Freiburg, Freiburg, Germany; 5 Institute for Cognitive and Affective Neuroscience, University of Trier, Trier, Germany

**Keywords:** Autism spectrum, Dual eye-tracking, Naturalistic gaze behavior, Social interaction

## Abstract

**Background:**

Previous eye-tracking research on autistic individuals has mostly examined the gaze behavior of one individual in response to social stimuli presented on a computer screen, suggesting that there is atypical gaze behavior. However, it is unknown how these findings translate to the interactive dynamics of gaze behavior during “face-to-face” encounters between two individuals. Only by analyzing the gaze behaviour of both interaction partners is it possible to determine the frequency of actual eye-contact and who initiates or breaks such periods of mutual eye gaze. The knowledge gained from this analysis could contribute to theorizing about the psychological mechanisms (e.g., gaze avoidance vs. gaze indifference) underlying autism.

**Methods:**

The present study applied a novel dual eye-tracking setup that allows the assessment and analysis of the interactive dynamics of gaze behavior regarding (i) mutual eye gaze (i.e., eye contact), (ii) initiations, and (iii) break-ups of eye contact. Participants (37 autistic individuals, 37 age- and IQ-matched neurotypical individuals) performed a semi-standardized social interaction (i.e., Fast Friends Procedure) with a confederate (trained to interact in a standardized manner).

**Results:**

Eye contact was reduced in interactions involving autistic individuals. Additional analyses revealed that this reduction was primarily due to the more frequent breaking of eye contact by these individuals. We also found considerable heterogeneity among autistic individuals, with atypical gaze behavior present in only about half of the sample.

**Limitations:**

Further research is required to determine whether the interactive dynamics of gaze behavior observed in this dual eye-tracking setup can be generalized to real-world situations. Future studies could also include arousal-related physiological measures.

**Conclusions:**

By tracking the gaze behavior of two interacting individuals, this study reveals specific atypicalities in the interactive dynamics of gaze behavior in a subset of autistic individuals, potentially informing diagnostic and therapeutic decisions. More broadly, our study highlights the added value of dual eye-tracking in elucidating the interactive nature of social encounters in both neurodiverse and neurotypical individuals.

**Trial registration:**

The study was registered as a clinical trial before starting data collection (https://drks.de/search/en/trial/DRKS00018957; Registration Date: 12/17/2019).

**Supplementary Information:**

The online version contains supplementary material available at 10.1186/s13229-025-00645-5.

## Background

**Fig. 1:**
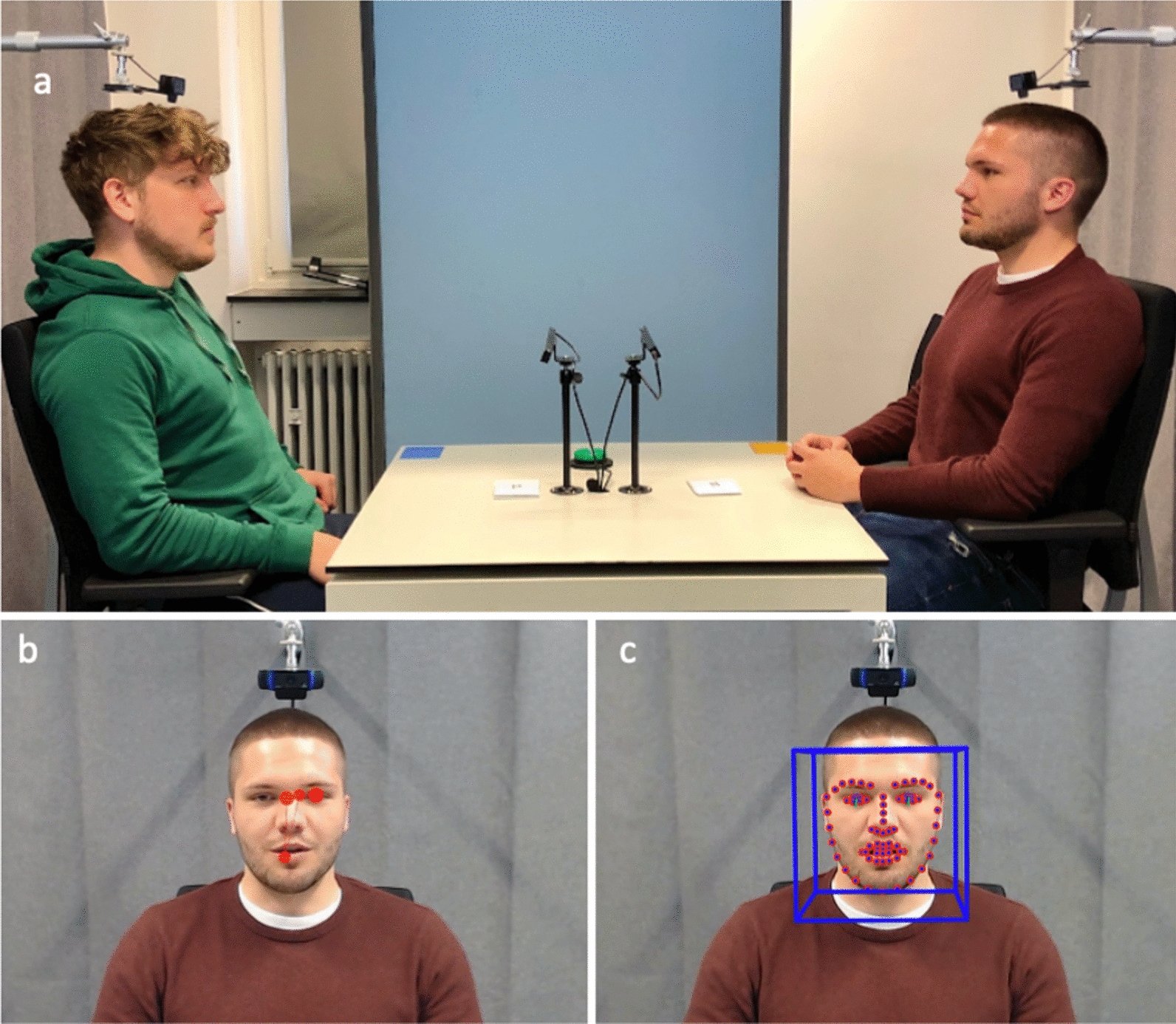
*Note*. The experimental dual eye-tracking setup in which both individuals face each other with the remote eye-tracking devices placed in the middle of the table and two cameras above each head recording the participants’ field of view (visualisation after [15]). **a** Example frame of the left individual’s gaze behavior on the facial features of the individual sitting opposite **b** and example frame for the OpenFace analysis of the right individual’s facial features **c**. We obtained informed consent from all displayed individuals for publishing identifiable images

Imagine a scenario where two individuals engage in a conversation, discussing their interests and sharing personal stories. As they exchange words, their eyes both receive and send pivotal social information (e.g., turn-taking, listening, signalling social interest, [1, 2]), enabling them to continually monitor and dynamically adapt their behavior depending on their interaction partner’s behavior [3, 4]. This enables humans to establish smooth and reciprocal communication during social encounters. However, for autistic individuals, this fundamental aspect of human interaction often presents challenges, raising questions about how their atypical gaze behavior [5, 6] impacts the dynamics of face-to-face exchanges. Although research on gaze behavior in autism has yielded less consistent findings than we might expect from clinical observations, at least in adult populations there is evidence that autistic individuals tend to exhibit briefer fixation duration towards the eyes and face in general and longer fixation duration towards non-social regions [7–10]. However, the vast majority of these studies were conducted in highly standardized laboratory settings involving the passive observation of social stimuli (e.g., faces, videos) presented on a computer screen, with no actual social interaction taking place. A few pioneering studies have investigated gaze behavior of autistic individuals in natural social interactions [11–14], but none of these studies applied dual eye-tracking to illuminate the interactive nature of gaze behavior in two individuals. It is therefore still unclear whether the atypicalities observed in “face-to-screen” experiments generalize to more ecologically valid “face-to-face” social interactions. Most importantly, the question of which aspects of gaze behavior’s interactive dynamics (e.g., initiations of eye contact, break-ups of eye contact) might be altered in autistic individuals remains unanswered. Capitalizing on a novel dual eye-tracking setup with no devices interfering with natural eye contact (see Fig. 1), we aimed to compare these interactive dynamics across autistic individuals and an age- and IQ-matched sample of neurotypical individuals.

It is essential to investigate interactive gaze behavior while two individuals are interacting socially [3, 4, 8]. First, another individual’s actual physical presence creates a social setting fundamentally different from passively viewing social stimuli while sitting alone in front of a computer screen. Actually meeting the other individual might trigger somewhat higher emotional arousal due to the awareness of how the other individual might evaluate one’s own behavior and how one should behave in such a social situation. Second, interacting individuals communicate reciprocally with each other via their gaze behavior. Tracking the gaze behavior of two interacting individuals provides new opportunities to analyze the interactive dynamics of gaze behavior characteristics, opportunities that are not available in studies tracking only a single individual’s gaze behavior. Even if one assesses the eye gaze of one individual who is interacting with another individual [11–14], we gain no knowledge about the interaction partner’s gaze behavior, meaning that the interactive dynamics of gaze behavior (e.g., who initiates or breaks eye contact) remain a “black box”. In contrast, dual eye-tracking reveals unique information on how long two individuals hold eye contact (i.e., display mutual eye gaze), and on which of the two interacting individuals is initiating or breaking-up eye contact [15]. Revealing which of these interactive dynamics might be altered in autistic individuals could inform our understanding of the psychological mechanisms underlying autism.

Theoretical accounts propose two main psychological mechanisms underlying atypical gaze behavior associated with autism. On the one hand, the “gaze avoidance account” suggests that autistic individuals actively avoid eye regions to minimize hyperarousal caused by the experienced threat evoked by another individual’s direct gaze [16–19]. On the other hand, the “gaze indifference account” explains atypical gaze behavior via an diminished propensity to direct attention to salient social stimuli like the eyes and faces [20–22]. While both models would predict reduced eye contact in interactions involving autistic individuals, they might also be associated with specific atypicalities of interactive gaze behavior: if autistic individuals experience eye contact as aversive, they should be especially apt to break up eye contact more often. This is because it seems difficult to avoid eye contact from the start in anticipating the aversive arousal, given that attention seems to be spontaneously biased towards faces and eyes [23, 24]. On the other hand, if autistic individuals are indifferent to eye gaze, they in particular should tend to initiate eye contact less often, because the eyes are not perceived as engaging. Once attention has been directed towards the eyes and eye contact has been established, we do not assume that gaze indifference would result in higher probability to break up eye contact [20]. In sum, analyzing interactive gaze behavior has the potential to shed some light on the psychological mechanisms of atypical gaze behavior in autistic individuals during naturalistic face-to-face interactions.

The present study illuminates the interactive dynamics of gaze behavior in autistic individuals during a naturalistic social encounter from “face-to-face”. For that purpose, we applied a dual eye-tracking setup that enables the assessment of gaze behavior from two individuals at the same time, while ensuring eye-tracking accuracy within an experimentally controlled, laboratory setting [15, 25]. We compared gaze behavior during a semi-standardized social interaction across autistic individuals and age-, and IQ-matched neurotypical individuals. This interaction involved the Fast Friends Procedure, which simulates a social encounter among strangers through escalating, mutual self-disclosure in a series of turn-taking questions [15, 26]. This procedure also enables us to check whether gaze behavior differs between talking and listening situations. As eye gaze is known to be more intense when listening than when talking [2, 15], we hypothesize that potential atypicalities in interactive gaze behavior are especially pronounced in this social role. To reduce the complexity of this experimental design, all participants interacted with a same-sex confederate blind to their interaction partner’s group and trained to interact and provide eye contact in a standardized manner. We analyzed both one-directional (e.g., unilateral gaze; see Methods for details) and interactive gaze parameters (e.g., eye contact, initiations, and break-ups of eye contact; see Methods for details) and also tested for the effect of “listening” vs. “speaking”. Controlling for the confederate’s gaze behavior, we thereby aimed to provide insights into which specific atypicalities of the interactive dynamics of gaze behavior (e.g., fewer initiations of eye contact, more break-ups of eye contact) may underlie the hypothetical reduced eye contact during naturalistic, social interactions in autistic individuals. We also aimed to illuminate sources of heterogeneity within autism [18, 27].

## Methods

### Participants

The study was approved by the local Ethics Committee (Approval ID: 439/15) and registered as a clinical trial before starting data collection (https://drks.de/search/en/trial/DRKS00018957). Please note that this study was originally planned to be part of a dual-center project aimed at assessing interactive gaze behavior in autistic individuals and social phobia. Due to differences in hygiene and safety regulations during the Covid-19 pandemic in the two involved universities located in two different federal states, data at the two locations were collected independently from each other. As there are higher prevalence of autism in males [28] and potential phenotypical differences across male and female autistic individuals [29, 30] which could not be addressed within this study’s framework, this study focused on male participants (see also Limitations). Thirty-nine adult male autistic individuals (according to the Diagnostic and Statistical Manual of Mental Disorders (DSM-5) [5], and confirmed by several clinical instruments, see Psychometric Measures and Clinical Instruments) and 39 neurotypical male individuals case-matched for age and total IQ (see Table 1) took part in the experiment. On the basis of a recent meta-analysis suggesting an effect of atypical gaze behavior with g = 0.47–0.50 (Hedges g) for socially complex interactions [9], we conducted an a priori power analyses (using G*Power, version 3.1.9.4, [31]). With a significance criterion of α = 0.05 and power = 0.80, the minimum total sample size needed with this effect size is N = 65. We investigated 78 participants (39 per group) to account for potential drop-outs during the study procedure. Participants were recruited at the Department of Psychiatry and Psychotherapy of the University Medical Center Freiburg, via flyers, and announcements on an institutional notice board and in the local newspaper. To control for any mating effects [32, 33], we included only heterosexually-oriented participants. All participants underwent telephone screening to monitor whether they fulfilled our inclusion criteria: taking no medication, normal to low corrected-to-normal vision (≤ 3 diopters), no other psychiatric condition, no current psychotherapy (for details, see Table S1). Based on data from a simulation study on the applied dual eye-tracking setup [25], we defined cut-offs for sufficient data quality (low quality: accuracy > 1.0° and precision > 0.5°). These cut-offs resulted in the exclusion of two autistic individuals (and the two matched neurotypical individuals). Note that data quality did not differ across groups (*all Fs (1, 74)* ≤ *. 1.44, p* ≥ *0.234, η*^2^ ≤ *. 0.009,* for details see Table S2b) and that including the outliers in the analysis led to similar findings. Our final sample consisted of n = 37 autistic individuals and n = 37 neurotypical individuals (for descriptive statistics and psychometric measures see Table 1). All participants gave written informed consent before the experiment and received €75.00 for their participation.

**Table 1:** Descriptive parameters and psychometric measures for autism and neurotypical groups

	Autism		Neurotypical			
	*M*	*SD*	*M*	*SD*	*F*(1, 74)	*p*
	n = 37		n = 37			
Age	33.59	11.37	33.35	10.97	0.01	.938
IQ (crystalline)	115.78	16.67	115.16	13.59	0.03	.860
IQ (verbal)	106.41	6.36	107.68	5.78	0.81	.372
ADOS-2	10.70	2.75	0.70	1.61	364.47	< .001
Com	3.97	1.48	0.32	0.78	175.43	< .001
Soc. Int	6.76	2.21	0.38	0.95	258.65	< .001
AQ	35.95	7.66	14.95	5.73	184.82	< .001

Mean (*M*) and standard deviation (*SD*). IQ (crystalline): Culture Fair Test (CFT), IQ (verbal): Verbal Intelligence Test (WST), ADOS: Autism Diagnostic Observation Schedule-2 with subscales Communication (Com.) and Social Interaction (Soc. Int.), AQ: Autism-Spectrum-Quotient

### Setup

The experiment took place in a bright room with constant artificial lighting, a white table (size: 80 cm × 80 cm; height: 72 cm) and two identical and height-adjustable chairs. Chairs could be replaced by a movable vertical calibration wall (180 × 80 cm, nine black crosshairs with a total radius of 3.5 cm and 1.5° visual angle on a white background resulting in a calibration area of 40 × 40 cm and 17.3° × 17.3° visual angle). The dual eye-tracking setup consisted of two Tobii X3 remote infrared eye-trackers (sampling frequency: 120 Hz) and two cameras (Logitech C920; FHD resolution: 1920 × 1080 px with 30fps) arranged to track the gaze behavior of two participants sitting across from each other at a table (for detailed information, see [15, 25]). The two eye trackers were placed on the table and the two cameras were mounted above the participants’ heads. Compared to Tönsing et al. [15], there were only minor changes in distances and angles (angle of remote eye-tracking devices: 28°; height of both scene cameras: 114 cm). Due to the restrictions related to the Covid-19 pandemic, it was necessary to increase the distance between the participant and confederate from 131 to 151 cm for half of the experiment (eye-tracking data quality, one-directional and interactive gaze behavior were not affected, for more details see Tables S2, S3 and S4). A movable green push-button used to synchronize the two eye-tracking data streams and segment the interaction was placed in the middle of the table. See Fig. 1 for further details and sample images and Videos S1-2 for illustration.

### The Fast Friends Procedure

The Fast-Friends-Procedure (FFP) scaffolds semi-standardized communication and builds rapport between unknown others through mutual self-disclosure in a series of turn-taking questions [26]. In line with Tönsing et al. [15], we used 12 questions from the original FFP translated into German (e.g., "What would constitute a perfect day for you?"; see Table S5 for details.).

### Psychometric measures and clinical instruments

To screen for a wide range of psychopathological characteristics, the Mini-Symptom Check List (Mini-SCL, [34, 35]) was completed by participants. Depressive symptoms were assessed using the Beck Depression Inventory (BDI-II, [36, 37]). Autistic characteristics were assessed via the Autism Spectrum Quotient (AQ, [38, 39]). Empathy was assessed using the Interpersonal Reactivity Index (IRI, [40, 41]). Social anxiety was quantified with the Social Interaction Anxiety Scale (SIAS-D, [42, 43]). Subjectively experienced fear and avoidance of eye contact were assessed with the Gaze Anxiety Rating Scale (GARS, [44, 45]). Fearful or worrying cognitions were measured using the Brief Fear of Negative Evaluation (BFNE, [46, 47]). Furthermore, we administered the structured observation instrument Autism Diagnostic Observation Schedule 2 module 4 (ADOS-2, [48, 49]) and Structured Clinical Interview for DSM-5 Disorders-Clinician Version (SCID-5-CV, [50, 51]). We also assessed the revised Culture Fair Test (CFT-20-R, [52, 53]). To measure verbal intelligence, we included the Multiple Choice Vocabulary Test (Wortschatztest, WST, [54]).

### Subjective ratings of interaction quality

Participants responded to six items on a visual analog scale (VAS, 1 = *not at all*, 100 = *very much*) before and after the interaction. These items assessed shame, anxiety, satisfaction, desire to escape, desire for support, and stress. After the interaction, we presented further items on attractiveness, authenticity, liking, sympathy, own enjoyment, distraction, desire to continue, laughter, role enjoyment, perceived self-disclosure of the other, one’s own openness, and the interaction partner’s openness on the same VAS (for full items see Table S6). We also used the Inclusion of Other in the Self Scale (IOS, [55]) to assess perceived interpersonal closeness (pair of circles to choose from; 1 = *no overlap* to 7 = *most overlap*).

### Confederates

To ensure comparable interactions for all participants, they interacted with one of six same-sex confederates (age: *M* = 27.33, *SD* = 4.03) who were blind to their interaction partner’s group and who were trained to interact in a standardized manner. All confederates should demonstrate an average amount of gaze and facial expression behavior. Furthermore, we took care that the number of experimental sessions with autistic individuals and with neurotypical individuals was counterbalanced for each confederate. They should answer the questions in approximately 30 s (120 s for the last question). All answers should be of the same valence, structure and length; only personal details were adapted for the confederates. Confederates were further trained to adhere to the experimental procedure as closely as possible (e.g., by compensating for too short and too long answers, reacting confidently to unforeseen events and staying on schedule; see Experimental Procedure for more details). We, authors of this manuscript, performed the training and provided detailed video feedback until all requirements for the confederates’ behavior were fulfilled (two confederates had to be excluded because they were unable to behave consistently as trained).

### Experimental procedure

Interested participants first completed a short telephone screening (10 min) to check the general inclusion criteria (for more details see Table S1). They were then invited to attend the diagnostic session conducted by a trained and experienced psychologist. Participants first gave informed consent to take part in this study, and to record the diagnostic session and experiment for study purposes. The interviewer administered the CFT-20, SCID-5, and ADOS to all participants. This was followed by the aforementioned psychometric measures, which were completed on a tablet computer. Participants could take a break in between. The entire diagnostic session lasted approximately 2.5 h. Autistic individuals were included in the study if they met the DSM-5 criteria for autism [5] and did not meet the DSM-5 criteria for another psychiatric condition. Of 57 autistic individuals invited to the first appointment, 16 were excluded for failing to meet our criteria (two further autistic individuals did not appear to the second appointment). Neurotypical individuals had to be free of any psychiatric condition (for more details see Table S1).

At the experimental session, participants were first instructed to take part in a social interaction task with a second person (confederate), during which they had to ask and answer prepared questions (FFP). If the participant and confederate were already acquainted, a new appointment was arranged with a different confederate. First, the experimenter brought the confederate (supposedly chosen at random) into the experimental room where the setup (e.g., seat height, chair position) was optimized for the eye-tracking recording. The eye-tracking calibration and validation procedure was then completed (see [15] for details). The participant was waiting in another room during this procedure. After successful calibration, the same procedure was repeated with the participant. When both were seated again, we conducted a final validation procedure in which both participants looked at their opponent’s distinctive facial features (e.g., left eye, right eye, nose, mouth). The experimenter then read a standardized introduction to the social interaction task (FFP) and then left the room. This procedure was first carried out with a practice trial. The confederate read the first question printed on a card, and the participant answered the question first. He had approximately 30 s to answer and was instructed to press a green button before starting to speak. The confederate then pressed the button, answering the same question within 30 s. After 60 s, a beep signaled to both to move on to the next question. The interaction partners had to answer 11 questions with the same pattern of rotating pose-and-answer and time limit (due to its more open framing, the last question had a 120 s time frame for the participant and confederate). The interviews lasted 15 min, and when the dyad had finished the last question, they rang a bell and the experimenter returned to the room. A further facial feature validation procedure was carried out for both participants. For the final part of the experiment, the participant and confederate completed subjective rating items on two tablet computers. After a short break, the participants also performed tasks including screen-based eye-tracking, which we will analyze elsewhere. They were then compensated for their expenses and seen off. In total, the laboratory experiment lasted approximately 2.25 h.

#### Data analysis

##### Preprocessing

All analyses were based on averaged binocular data. For eye-tracking validation, segments of eye-tracking data were checked for saccades. In cases of a saccade (e.g., the marker was set before the participant fixated on the cued point), the markers were adjusted (for more details see [25]).

##### Accuracy, precision, and robustness

Accuracy is defined as the mean offset of the gaze point position relative to the target point position and is reported in degrees of gaze angle. Precision is calculated as a measure of the variance (SD) of data samples in degrees. Robustness includes the percentage of valid eye-tracking data points for a given time sequence. Analyses of the eye-tracking data quality indices were performed using in-house scripts in Matlab (R2018a, version 9.7.0). As can be seen in Table 2, the eye-tracking data quality indices were on a very high level and comparable to previous studies with this setup [15, 25].

**Table 2:** Eye-tracking data quality for the validation sequences (wall, face pre & face post)

	Wall	Face (pre)	Face (post)	Total
	*M* (*SD*)	*M* (*SD*)	*M* (*SD*)	*M* (*SD*)
Accuracy	0.42° (0.18°)	0.52° (0.19°)	0.58° (0.22°)	0.50° (0.13°)
Precision (*SD*)	0.35° (0.11°)	0.38° (0.14°)	0.37° (0.11°)	0.37° (0.08°)
Robustness	98.78% (2.84%)	98.45% (5.48%)	98.87% (4.45%)	98.70% (3.06%)

Mean (M) and standard deviation (SD) for eye-tracking quality are shown for all measurement points (Face pre, Face post). Accuracy and precision are given in visual angle and robustness in percent. Precision (SD): Precision is calculated as the standard deviation of the data sample. Of the 3696 tracks, 189 markers (5.11%) had to be corrected due to saccades. 204 markers (5.52%) had to be shifted due to suboptimal marker timing. 79 trials (2.14%) had to be excluded due to experimenter error. 37 trials (1.00%) had to be excluded due to robustness < 80.00%

##### Definition of areas of interest

In line with Tönsing et al. [15], we assessed the facial landmark detection tool OpenFace [56] by analyzing the scene videos to generate facial landmarks. The limited-radius Voronoi tessellation method (radius of 2° for 131 cm distance and 1.73° for 151 cm; [57]) was applied to obtain the areas of interest (AOI) eyes (right and left eyes added together), nose, mouth and the rest of the face [25, 58]. An ellipse around the face provided the AOI face.

##### Analysis of one-directional eye-tracking data

Total dwell-time on a given AOI was defined as the percentage of total frames on the given AOI (e.g., eyes or nose) within a given segment including all gaze behaviors (fixations, saccades, etc.). The percentage of missing data was also calculated including all frames with no data due to gaze outside the calibration area or loss of eye-tracking. For the one-directional gaze analysis, we calculated the average time spent looking at the other person’s eyes as a percentage of total time. We did so for all trials separately, while differentiating between speaking and listening phases, as well as aggregating over both.

##### Analysis of interactive eye-tracking data

For the analysis of interactive eye-tracking data, we first had to synchronize the two gaze data streams in time and we then classified specific events in that data. Initiations were defined as events where initially both individuals are not looking at each other, and then one individual starts to gaze at the opponent’s eyes for at least 200 ms. The threshold for initiating eye contact was set at 200 ms [15], as this represents the human reaction time to visual stimuli [59, 60]. This threshold was chosen to minimize the number of very short and any random events potentially introducing noise to subsequent analyses. However, different thresholds (0 ms to 1000 ms) revealed similar result patterns (see Table S7). Mutual eye gaze was defined as events where both participants gaze at the opponent’s eyes (we also analyzed mutual face gaze, i.e. when both participants gaze at the opponent’s face, for details see Tables S14 and 15). Finally, we identified break-ups, as events where one individual terminated eye contact. The number of initiations/break-ups was compared to the number of initiations/break-ups by the counterpart, resulting in an initiation/break-up ratio given in percentages. The total duration of mutual eye gaze was related to the duration of the total conversation as well, resulting in mutual eye gaze given as a percentage of total time.

#### Statistical analysis

Statistical analyses were performed using SPSS (28th edition), RStudio (RStudio version 1.4.1106, R version 4.2.1), and MATLAB (version 2018b) software. The analyses of Bayesian statistics were carried out in JASP (version 0.16.3, 202,161). All tests were performed as two-tailed tests with a significance level of alpha = 0.05. In cases of multiple testing or post hoc testing, Bonferroni correction was applied. In the case of heterogeneity of covariance (Mauchly sphericity test), Greenhouse–Geisser corrections were applied. The Shapiro–Wilk test indicated non-normal distribution of the one-directional and interactive gaze data variables (all *Ws* ≥ 0.83, *ps* ≤ 0.001).

For the one-directional and interactive eye-tracking data, we calculated the intraclass correlation (ICC), as a considerable amount of variance could be explained by clusters of dependent data structure (mutual eye gaze: ICC = 0.67). In fact, groups were not independent as they were interacting with the same confederates. Therefore, and as one-directional and interactive eye-tracking data were not normally distributed, we conducted generalized linear mixed models for these variables (GLM, package *lme4* in R; [61]). First, to identify the random slope structure with the best fit, models with the full fixed-effects structure were tested. The full fixed-effects models with different random slopes were compared using the Akaike Information Criterion (AIC; [62, 63]). With the optimized random-effects structure, fixed predictors were added to the model step by step, compared, keeping only those effects that significantly improved the predictor model. We applied a z-standardization to all metric variables to provide interpretable model coefficients for GLM. For dependent variables, a generalized linear mixed model with questions and phase (Level 1), nested in group, confederates, distance, and participants (Level 2) was conducted. We tested for effects of group (autistic individuals vs. neurotypical individuals), questions (1 to 11), and phase (speaking or listening). We also checked whether psychometric measures improved the model fit as fixed effects. As random effects, we defined subjects, confederates, and the distance between individuals. We included by-participant slopes for all fixed effects and random intercepts for all possible predictors, adhering to standard guidelines for linear mixed-effects modelling [64].

For comparisons of the autism subgroups (between-subjects factor *group*) we ran MANOVAs [65]. Bayesian analyses were performed using the Bayesian *t*-test approach, as non-significant results applying frequentist statistics do not mean that the alternative hypothesis is true [66, 67]. The null hypothesis states that the two groups do not differ in gaze behavior H0: δ = 0. The two-tailed alternative hypothesis (H1) states that the two groups differ from each other. The default prior option provided by JASP was used to assess the likelihood of the alternative hypothesis given the data (BF10) compared to the null hypothesis. The Bayes factor may indicate higher likelihood for one of the hypotheses.

## Results

### Confederate behavior and ratings

In the first step, we checked whether confederates indeed were able to exhibit standardized gaze behavior when interacting with autistic individuals and neurotypical individuals (factor group). Regarding total dwell-time, there was no significant effect of group for any AOI (all *F*(1, 74) ≤ 5.48, *p* ≥ 0.110, *η*^2^ ≤ 0.071). The same pattern appeared for total fixation time, mean fixation duration, and total number of fixations (all *F*(1, 74) ≤ 1.92, *p* ≥ 0.092, *η*^*2*^ ≤ 0.039; for more details see Table S8).

We also analyzed the confederates’ ratings regarding interaction quality. There was no effect of group for items rated pre and post (all *F*(1, 74) ≤ 1.15, *p* ≥ 0.286, *η*^*2*^ ≤ 0.016). Confederates did not differ in rated and desired interpersonal closeness in regard to interacting with autistic individuals or with neurotypical individuals (all *F*(1, 74) ≤ 2.17, *p* ≥ 0.145, *η*^*2*^ ≤ 0.028; for more details see Table S9).

### One-directional gaze behavior

**Fig. 2:**
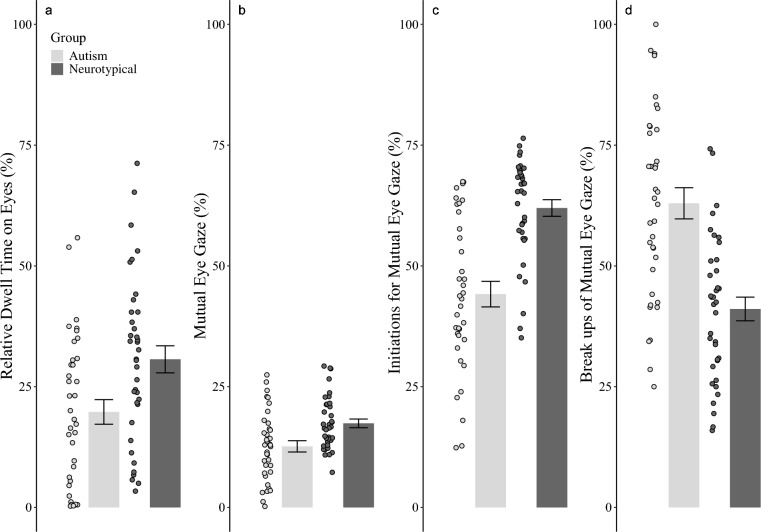
Gaze behavior parameter (one-directional and interactive) time for autistic individuals and neurotypical individuals. *Note*. **a** Total dwell-time on the eyes during the interaction as a percentage of total time, **b** mutual eye gaze during the interaction as a percentage of total time, **c** initiations for mutual eye gaze as a percentage of the total number of initiations, and **d** break-ups of mutual eye gaze as a percentage of the total number of break-ups by the participant and his interaction partner. Group mean is displayed with bars, standard errors, and individual means (black dot) for each participant

We first tested whether autistic individuals (compared to neurotypical individuals) gazed less towards facial features and sought any other differences in one-directional gaze behavior (fixations, number of fixations, mean fixation time). For the AOI total dwell-time on the eyes, the generalized linear mixed model with the best fit for the dependent variable total dwell-time on the AOI face contained random slopes for *trial* (Δ AIC: 191.42) and *phase* (Δ AIC: 6.65). Additionally, the model contained a fixed effect of *group* (*b* = 0.68, *SD* = 0.18, *t*(74) = 251.24, *p* < 0.001; Δ AIC: 231.51) with lower dwell-time levels on the face in autistic individuals (see Fig. 2a). Furthermore, the best model contained a fixed effect of *phase* (*b* = 0.04, *SD* = 0.09, *t*(74) = 21.27, *p* < 0.001; Δ AIC: 6.26) with higher dwell-time levels on the face during listening phases (for details see Tables S10 and 11). The interaction effect of group*phase was significant (*t*(74) = 23.66, *p* < 0.001) with more pronounced differences in dwell-time between speaking and listening phases in the neurotypical group (see Tables S10 and 11 for more details). There was no effect of *time* since the variable *time* did not improve the model. Likewise, the variable *confederate’s gaze behavior* did not become significant. Adding further variables (e.g., BDI, SIAS, IRI, BFNE) did not significantly improve the model fit (all *bs* ≤ 0.06, *SDs* ≤ 0.05, *ts*(74) ≤ 0.92, *ps* ≥ 0.361, Δ AICs ≤ 1.74). Models for AOIs nose, mouth, background and rest of face indicated similar effects (see Tables S10 and 11 for more details). We observed similar effects for the variables fixation time (see Table S12) and number of fixations (see Table S13) with shorter mean durations (*b* ≥ 0.54, *SD* ≥ 0.18, *t*(74) ≥ 10.05, *p* ≤ 0.001; Δ AIC: 12.05; see Tables S14 for more details) and fewer fixations (*b* ≥ 0.73, *SD* ≥ 0.22, *t*(74) ≥ 7.30, *p* ≤ 0.001; Δ AIC: 2.83; see Tables S14 for more details) in autistic individuals.

In summary, the analysis of one-directional eye-tracking data indicated differences in groups for several gaze parameters. Autistic individuals gazed less (dwell-time, fixation time, number of fixations) and shorter (fixation time) at facial features as eyes, mouth, and the total face.

### Interactive dynamics of gaze behavior

#### Eye contact

In the next step, we checked whether autistic individuals (compared to neurotypical individuals) engage in less eye contact with the confederate. The generalized linear mixed model with the best fit for the variable *mutual eye gaze* contained random slopes for *phase* (Δ AIC: 471.32). The best-fitting model contained a fixed effect of *group* (*b* = 0.31, *SD* = 0.05, *t*(74) = 56.16, *p* < 0.001; Δ AIC: 95.17) with lower levels of total mutual eye gaze for autistic individuals (see Fig. 2b). Furthermore, there was a fixed effect of *phase* (*b* = 0.36, *SD* = 0.08, *t*(74) = 13.11, *p* < 0.001; Δ AIC: 54.35) with higher levels of mutual eye gaze during listening phases. We detected no effect of *time*, indicating that mutual eye gaze did not change during the conversation (*b* = 0.05, *SD* = 0.06, *t*(74) = 0.35, *p* = 0.634). The model also contained a significant interaction effect group*phase (*b* = 0.26, *SD* = 0.06, *t*(74) = 9.15, *p* < 0.001; Δ AIC: 9.31). As in our unilateral analysis, the difference in mutual eye gaze between speaking and listening phases was less pronounced in autistic individuals than in neurotypical individuals (*t*(74) = 5.31, *p* < 0.001). Further variables did not significantly improve the model fit and were not included (e.g., BDI, SIAS, IRI, BFNE; all *bs* ≤ 0.28, *SDs* ≤ 0.11, *ts*(74) ≤ 0.80, *ps* ≥ 0.423, Δ AICs ≤ 0.83). We noted a similar result pattern in the total mutual face-gaze-duration variable (see Tables S15 and 16 for more details). In sum, we observed less eye contact during interactions involving autistic individuals.

#### Initiations and break-ups of eye contact

We tested whether autistic individuals (compared to neurotypical individuals) showed atypicalities in additional variables quantifying the interactive dynamics of gaze behavior (i.e., initiations and break-ups of eye contact). The generalized linear mixed model with the best fit for the dependent variable *initiations* contained a fixed effect of *group* (*b* = 0.15, *SD* = 0.11, *t*(74) = 54.24, *p* < 0.001; Δ AIC: 490.03) with lower initiation levels in autistic individuals (see Fig. 2c). Furthermore, the best-fitting model contained a fixed effect of *phase* (*b* = 0.40, *SD* = 0.08, *t*(74) = 14.05, *p* < 0.001; Δ AIC: 85.93) with higher levels of initiations during listening phases. The generalized linear mixed model with the best fit for the dependent variable *break-ups* contained a fixed effect of *group* (*b* = 0.25, *SD* = 0.13, *t*(74) = 34.74, *p* < 0.001; Δ AIC: 90.43) with higher levels of break-ups in autistic individuals (see Fig. 2d). Furthermore, the best-fitting model contained a fixed effect of *phase* (*b* = 0.34, *SD* = 0.08, *t*(74) = 12.47, *p* < 0.001; Δ AIC: 61.55) with higher levels of break-ups during listening phases. This model also contained a significant interaction effect group*phase (*b* = 0.39, *SD* = 0.08, *t*(74) = 7.21, *p* < 0.001; Δ AIC: 19.75). Autistic individuals interrupted eye contact particularly often when they were in the response role (see Tables S15 and S16 for more details).

In the next step, we checked whether the reduced eye contact in break-ups involving autistic individuals was driven by less initiations and/or more break-ups of eye contact. We conducted commonality analysis which decomposes the model’s *R*^2^ statistic (i.e., explained variance) for mutual eye gaze into commonality coefficients *R*^2^ for each participant and confederate event class. Break-ups of mutual gaze events by autistic individuals explained the most variance, R^2^ = 0.158. As the second variable, fewer initiations accounted for the second most variance, R^2^ = 0.076. Thus, the reduced mutual eye gaze was largely explained by the more frequent break-ups and, to a lesser degree, by fewer initiations of eye contact by autistic individuals.

In summary, the analysis of interactive eye gaze behavior demonstrated that autistic individuals have less eye contact with the confederate than neurotypical individuals. Additional analyses revealed that reduced eye contact was largely driven by more break-ups by autistic individuals.

### Subjective ratings of interaction quality

We also checked whether autistic individuals and neurotypical individuals differed in subjective ratings of interaction quality. For the six items participants rated pre and post, we calculated an ANOVA with repeated measurements. Before the interaction took place, the autism group delivered significant higher scores for “shame”, “fear”, “wish to leave” and “perceived stress”, and lower rates for “happiness” (all *F*(1, 74) ≥ 4.73, *p* ≤ 0.033, *η*^*2*^ ≥ 0.059; for more details see Table S16).

After the interaction took place, we noted significant effects with higher levels in the autism group for the items “fear”, “wish to leave” and “stress” and lower levels for the item “happiness” (all* F*(1, 74) ≥ 4.50, *p* ≤ 0.037, *η*^*2*^ ≥ 0.059). Furthermore, there were significant effects for time, with "perceived stress” showing lower scores after the interaction, and "fear" and “happiness” showing higher scores (all *F*(1, 74) ≥ 5.82, *p* ≤ 0.017, *η*^*2*^ ≥ 0.037). However, we detected no interaction effect of *group* and *time* for any of these items (all *Fs*(1, 74) ≤ 0.48, *p* ≥ 0.488, *η*^*2*^ ≤ 0.003; see Table S17 for more details). For further post items rated only once after the interaction, the autism group rated the items “enjoyment”, “enjoyment of their role”, and “perceived fun” significantly lower (all *F*(1, 74) ≥ 5.56, *p* ≤ 0.021, *η*^*2*^ ≥ 0.069, all other *F*(1, 74) ≤ 3.15, *p* ≥ 0.080, *η*^*2*^ ≤ 0.040; for more details see Table S17).

Finally, we analysed associations of the subjective ratings of interaction quality with actual gaze behavior. We found significant associations only for ratings after the interaction took place (pre-interaction ratings: all *rs* ≤ -0.249, all *ps* ≥ 0.138): Individuals who experienced more “stress”, “shame”, and “fear” displayed lower levels of mutual eye gaze (all *rs* ≥ -0.356, all *ps* ≤ 0.030; see Table S18 for more details); and individuals who experienced more “sympathy” and “fun” (all *rs* ≥ 0.434, all *ps* ≤ 0.007; see Table S18 for more details) displayed higher levels of mutual eye gaze. In summary, autistic individuals rated more negative as well as less positive emotions both before and after the social interaction took place, and post-interaction ratings correlated significantly with having eye contact.

### Subgroup analysis

Motivated by visual analysis of Fig. 2, we tested for variance homogeneity across the experimental group. We found evidence of heterogeneous variances across groups (all *Fs*(1, 74) ≥ 8.62, *ps* ≤ 0.004) for several gaze parameters with larger variance within autistic individuals. The 99% confidence interval for the dwell-time variable on the eyes of the neurotypical group CI [23.04, 38.30] included 17 autistic individuals, whereas only 20 individuals from this group exhibited eye gaze below the lower threshold of that interval (note that a two-step cluster analysis revealed exactly the same group assignment; for details see Supplementary Results). These findings suggest the existence of at least two subgroups within our sample of autistic individuals, one group showing gaze behavior indistinguishable from that of neurotypical individuals, and one subgroup revealing reduced eye gaze (for details, see Tables S19, S20. S21 and S22).

In the next step, we checked whether we could find any variables (psychometric measures, clinical instruments, and subjective ratings) differing across these two subgroups of autistic individuals (one “high autism” group with values within the CI from neurotypical individuals, and another “low autism” group with values below the lower CI threshold). Surprisingly, among all state, trait, subjective, and objective rating parameters, we observed that groups differed only in the perceived “shame”, with higher shame ratings in the “high autism” group (*F*(1, 74) = 5.41, *p* = 0.023, *η*^2^ = 0.013). For all other variables there was no difference between groups (all *F*(1, 74) ≤ 1.72, *p* ≥ 0.143, *η*^2^ ≤ 0.009; Bayes Factors ranged from 1.48 to 3.12 suggesting that the likelihood of the alternative hypothesis – stating a group difference—is only slightly, i.e. 1.48 to 3.12 higher than that of the null hypothesis; for more details see Table S19, S20, S21, and 22). This seems to indicate that there may be two subgroups of autistic individuals distinguishable only in their gaze behavior, but not in other parameters.

## Discussion

Relying on a novel dual eye-tracking setup, the present study compared the interactive dynamics of gaze behavior of autistic individuals to that of an age- and IQ-matched sample of neurotypical individuals during a naturalistic social encounter with a confederate (i.e., the FFP). As intended, the confederates revealed standardized gaze behavior that did not differ across groups. In contrast, we observed specific atypicalities in the interactive dynamics of gaze behavior among autistic individuals. Compared to neurotypical individuals, autistic individuals revealed fewer initiations and more break-ups of mutual eye gaze (i.e., eye contact), resulting in reduced eye contact with the confederate. Additional analyses suggested that reduced eye contact was mainly because autistic individuals broke-up eye contact more often. All differences across groups were more pronounced when participants were listening rather than talking during the social encounter, suggesting specific communication changes in autism. Autistic individuals also experienced the conversation as more aversive than neurotypical individuals. Overall, this study reveals several atypicalities in interactive gaze behavior in autism. Given the key role of eye contact in coordinating attention, understanding the other and exchanging information in social encounters (for example by signaling passes of the conversational baton; [68–70]), our findings might yield novel insights into the psychological mechanisms underlying altered social interaction associated with autism.

Specifically, our study reveals that reduced eye contact in interactions involving autistic individuals was mainly driven by more frequent eye contact break-ups by these individuals. Analyses of subjective ratings suggested that autistic individuals were less able or willing to maintain mutual eye gaze because they experience this state as somewhat aversive. These findings could be interpreted as favoring the “gaze avoidance account”, explaining atypical gaze behavior in autism by the attempt to minimize gaze-related hyperarousal [16–19]. Future studies including physiological measures of arousal (e.g., heart rate, [71, 72]) or even experimentally manipulating a situation’s aversiveness could well reinforce this assumption. On the other hand, we also observed that autistic individuals initiated eye contact less often, although this effect contributed less to the overall reduced eye contact. This finding also provides some evidence of the validity of the “gaze indifference account” explaining atypical gaze behavior by the inability or unwillingness to focus on the eyes as a source of social information [20–22]. It is interesting to relate these findings to another study applying dual eye-tracking during social observation via videoconference, which demonstrated that two interacting individuals showing some autistic characteristics revealed decreased eye gaze only when the other individual was looking back (but more eye gaze when the other individual was not looking back; [73]). They argue that these findings also provide evidence favoring the “gaze avoidance account” rather than the “gaze indifference account”, as eye gaze should not depend on the other individual’s eye gaze if individuals are indifferent to eye gaze. Overall, the existing dual eye-tracking studies on the association of autism and eye gaze suggest that the eye contact avoidance shown by autistic individuals is at least partly due to the fact that they experience eye contact as aversive.

Additional mechanistic insights into gaze behavior in autism are provided by comparing eye gaze across listening and speaking phases. Autistic individuals displayed larger atypicalities in the listener role. As in previous studies [68, 69] we found that, in general, individuals show more eye gaze when listening rather than speaking. This pattern suggests that gazing towards the interaction partner’s eyes while listening seems to benefit functional communication, for example by reinforcing one’s attention or by receiving additional information from the speaker (e.g., when have they finished and when am I expected to take over, emphasizing essential parts of communication by making eye contact while speaking; [68, 69]). In contrast, establishing eye gaze while speaking may be somewhat limited in all individuals, as it requires additional cognitive demands that might instead be channelled to optimize one’s own communication [2]. The observation that atypicalities in autistic individuals are more pronounced when listening might indicate that they have fewer opportunities to both signal their own attention to the other as well as receive rich social information that facilitates understanding of their interaction partner. It also raises the question of whether autistic individuals need more support during multimodal processing to process social information across various channels during face-to-face interactions [74].

Besides providing evidence in favor of differential gaze behavior across the groups of autistics individuals and neurotypical individuals, the present study also directs our attention to considerable heterogeneity within the sample of autistic individuals. Strikingly, our subgroup analysis revealed that only one specific autism subgroup exhibited atypical gaze behavior, constituting approximately half of the sample of autistic individuals. In contrast, another autism subgroup’s gaze behavior was barely distinguishable from that of neurotypical individuals. Except for revealing higher ratings for shame, the autism subgroup that exhibited atypical gaze behavior did not differ from the autism subgroup that exhibited typical gaze behavior in various state and trait parameters (e.g., subjective ratings, measures of neurodivergence, ADOS data). This pattern is reminiscent of findings from screen-based, non-interactive eye-tracking [75] and neuroimaging studies [76] that also identified behavioral and biological atypicalities only in a subgroup of autistic individuals. Such atypicalities might reflect endophenotypes reflecting genetic effects [77], potentially serving as biomarkers for use in identifying autistic characteristics and providing support to autistic individuals [27, 78, 79]. Indeed, assessing interactive gaze behavior via the present dual eye-tracking setup has potential translational significance. For example, in the future one could build a database of normative interactive gaze behavior to see whether an individual reveals atypicalities (i.e., scores outside the 95% confidence interval of the sample population) in different aspects of interactive gaze behavior (i.e., initiating eye-contact, breaking-up eye contact) as well as underlying physiological responses (i.e., heart rate). Such findings could reveal additional diagnostic information and tailor support of autistic individuals [80, 81] by indicating which individuals might benefit from support modules focused on psychotherapeutically modulating specific aspects of eye contact [82, 83], which individuals might benefit from reducing their autonomic hyperarousal during social interaction [84, 85], and which individuals might benefit from changing cognitive distortions regarding their otherwise typical social behavior (e.g., “I am unable to make adequate eye contact”).

### Limitations

Whereas our study has taken an initial approach to analyze the interactive dynamics of gaze behavior based on event classification, our data might offer even more to discover when taking more complex analysis approaches (e.g., time series analysis; [86]). In addition, it would be insightful to add arousal-related physiological measures (e.g., heart rate or electrodermal activity) to check whether the typical interactive gaze behavior displayed by a subgroup of autistic individuals comes at a cost to them (i.e., accompanied by increased arousal). It is also an interesting question whether autistic individuals would show the same pattern of gaze behavior when interacting with other autistic individuals. According to the theory of the “double empathy problem” [87, 88], neurotypical individuals have difficulty understanding autistic individuals, meaning that the atypical gaze behavior of autistic individuals could also be driven by the “atypical” gaze behavior of neurotypical individuals. Future research should also include female autistic individuals to test the generalizability of the present study’s findings across genders. Finally, although our setup takes a large step forward in studying naturalistic, interactive social behavior under controlled experimental conditions, it remains a target of investigation as to whether the gaze behavior displayed within this setup generalizes to real-world gaze behavior, given that both the setup itself might still make participants aware of the test situation and the interaction was semi-structured.

## Conclusion

In sum, by exploiting analyses of dual eye-tracking data, the present study has identified specific atypicalities in the interactive dynamics of gaze behavior in autistic individuals' naturalistic face-to-face interactions. By identifying subgroups with either typical or atypical gaze behavior, we might be able to develop more individualized diagnostic and support approaches in the future. Furthermore, the present study raises issues ideal for future research. Are there any other psychological or biological parameters distinguishing gaze behavior subgroups? Do autistic individuals also exhibit altered interactive gaze behavior dynamics in social situations involving explicitly negative social evaluations or conflict [89]? Could administering oxytocin modulate interactive gaze behavior in autistic individuals who do not initiate but rather break-up eye contact [11, 90–92]? Overall, given the extensive significance of eye contact in enabling functional face-to-face social interactions in both neurotypical and neurodiverse individuals, this study’s minimally invasive dual eye-tracking setup could prove to be of great value for researchers across the social, clinical, and behavioral sciences.

## Supplementary Information

Additional file 1

Additional file 2

Additional file 3

## Data Availability

Data and materials are available on request.

## Declarations

### Autism-related language preferences

The authors of this paper aimed to use terminology that aligns with the language preferences of autistic adults, referring to [93].
