# Supplementary material for: Altered interactive dynamics of gaze behavior during face-to-face interaction in autistic individuals: a dual eye-tracking study
Source: Mol Autism. 2025 Feb 22;16:12. doi: 10.1186/s13229-025-00645-5 (PMC11846317; doi:10.1186/s13229-025-00645-5)
Supplement: Supplementary file 1 — Additional file 1 [file 13229_2025_645_MOESM1_ESM.docx]

- Supplementary Material –

Altered interactive dynamics of gaze behavior during face-to-face interaction in autistic individuals: a dual eye-tracking study

Daniel Tönsing*^x1^, Bastian Schiller* ^x 1,2^, Antonia Vehlen^3^, Kathrin Nickel^4^, Ludger Tebartz van Elst^4^, Gregor Domes^3,5^, and Markus Heinrichs ^x 1,2^

^1^Department of Psychology, Laboratory for Biological Psychology, Clinical Psychology, and Psychotherapy, University of Freiburg, Freiburg, Germany

^2^Freiburg Brain Imaging Center, University Medical Center, University of Freiburg, Freiburg, Germany
^3^Department of Biological and Clinical Psychology, University of Trier, Trier, Germany

^4^Department of Psychiatry and Psychotherapy, Medical Center ­ University of Freiburg, Faculty of Medicine, University of Freiburg, Freiburg, Germany

^5^Institute for Cognitive and Affective Neuroscience, University of Trier, Trier, Germany

*shared first-authorship, ^x^corresponding authors

Mail adresses: Daniel Tönsing (daniel.toensing@unibas.ch), Bastian Schiller ([schiller@psychologie.uni-freiburg.de](mailto:schiller@psychologie.uni-freiburg.de)), Antonia Vehlen (vehlen@uni-trier.de), Gregor Domes (domes@uni-trier.de); Kathrin Nickel (kathrin.nickel@uniklinik-freiburg.de), Ludger Tebartz van Elst (tebartzvanelst@uniklinik-freiburg.de); Markus Heinrichs ([heinrichs@psychologie.uni-freiburg.de](mailto:heinrichs@psychologie.uni-freiburg.de)).

*Supplementary Results: Two-step cluster analysis for subgroup analysis*

To confirm the identification of subgroups within the sample of autistic individuals by means of the confidence interval criterion (see main manuscript), we performed a two-step cluster analysis on the dependent variable of dwell-time on the eyes. This analysis suggested two clusters (cluster 1: n = 17, M = 33.83; cluster 2: n = 20, M = 7.84) with an average silhouette value of 0.7 (indicating good cluster quality). Note that this cluster analysis resulted in the same group assignment of autistic individuals as using the confidence interval criterion.

*Table S1.*

Inclusion criteria in general and specific criteria for both groups.

General:

- Gender: Male
- Age: 18-60 years
- Fluent German
- visual impairments under 3.0 diopters
- no other type of prescriptive visual aid (astigmatism, strabismus)
- symptom burden (Mini Symptom-Checklist) less than 10
- depressive symptoms less than 10 according to the Beck Depression Inventory
- alcohol intake > 4x/week
- no intake of psychotropic drugs
- no intake of illegal drugs
- no severe psychiatric disorder (schizophrenia, bipolar disorder, personality disorder)
- no acute psychiatric disorder (any affective disorder, any anxiety disorder)

Autism Group:

- Diagnosis of Autism according to the Diagnostic and Statistical Manual of Mental Disorders-5
- Autism Diagnostic Observation Schedule-score of 7 or higher, Autism Quotient-score of 25 or higher
- No primary diagnosis of social anxiety disorder
- no current psychotherapy (or the trial participation was prior to treatment)

Neurotypical Group:

- No diagnosis of Autism according to the Diagnostic and Statistical Manual of Mental Disorders-5
- No diagnosis of Social Anxiety according to the Diagnostic and Statistical Manual of Mental Disorders-5
- no current psychotherapy

*Note.* Exclusion criteria were alcohol, nicotine or drug abuse, studying or having a degree in psychology or economics, current or previous history of psychiatric conditions, or insufficient fluency in the German language.

*Table S2a.*

Eye-tracking data quality for both distances between individuals

|  | **Distance** | |  | | |
| --- | --- | --- | --- | --- | --- |
|  | 131 cm | 151 cm |  | | |
|  | *M* (*SD*) | *M* (*SD*) | *F*(1, 74) | *p* | *η*² |
| **Accuracy (°)** |  |  |  |  |  |
| Wall | 0.41 (0.19) | 0.43 (0.18) | 0.34 | .562 | .005 |
| Face (pre) | 0.52 (0.22) | 0.51 (0.17) | 0.09 | .764 | < .001 |
| Face (post) | 0.55 (0.22) | 0.60 (0.23) | 0.96 | .330 | .013 |
| total | 0.50 (0.13) | 0.52 (0.12) | 0.48 | .492 | .007 |
| **Precision (°)** |  |  |  |  |  |
| Wall | 0.36 (0.11) | 0.34 (0.11) | 0.44 | .511 | .006 |
| Face (pre) | 0.38 (0.15) | 0.39 (0.12) | 0.15 | .703 | .002 |
| Face (post) | 0.37 (0.13) | 0.38 (0.10) | 0.08 | .774 | .001 |
| total | 0.37 (0.09) | 0.37 (0.07) | < 0.01 | .957 | < .001 |
| **Robustness (%)** |  |  |  |  |  |
| Wall | 98.77 (1.81) | 98.71 (2.47) | 0.02 | .893 | < .001 |
| Face (pre) | 98.18 (6.54) | 98.80 (3.74) | 0.23 | .631 | .003 |
| Face (post) | 98.30 (5.91) | 99.55 (1.63) | 1.48 | .228 | .020 |
| total | 98.40 (3.84) | 99.02 (1.77) | 0.70 | .407 | .010 |

*Note*. Mean (*M*), standard deviation (*SD*) for the distances 131 cm and 151cm between individuals, *F*-test results (*F*) for parametric testing and effect size (*η*²).

*Table S2b.*

Eye-tracking data quality for both groups

|  | **Autism** | | **Neurotypical** |  | | |
| --- | --- | --- | --- | --- | --- | --- |
|  | *M* (*SD*) | *M* (*SD*) | | *F*(1, 74) | *p* | *η*² |
| **Accuracy (°)** |  |  | |  |  |  |
| Wall | 0.42 (0.20) | 0.42 (0.17) | | 0.02 | .890 | < .001 |
| Face (pre) | 0.52 (0.20) | 0.51 (0.18) | | 0.16 | .695 | .002 |
| Face (post) | 0.56 (0.24) | 0.59 (0.20) | | 0.32 | .573 | .004 |
| total | 0.50 (0.13) | 0.51 (0.08) | | < 0.01 | .951 | < .001 |
| **Precision (°)** |  |  | |  |  |  |
| Wall | 0.33 (0.10) | 0.36 (0.12) | | 1.11 | .295 | .015 |
| Face (pre) | 0.38 (0.14) | 0.38 (0.14) | | < 0.01 | .961 | < .001 |
| Face (post) | 0.37 (0.12) | 0.37 (0.11) | | < 0.01 | .947 | < .001 |
| total | 0.36 (0.08) | 0.37 (0.08) | | 0.29 | .595 | .004 |
| **Robustness (%)** |  |  | |  |  |  |
| Wall | 98.60 (2.05) | 98.89 (2.24) | | 0.33 | .565 | .005 |
| Face (pre) | 98.37 (6.29) | 98.57 (4.25) | | 0.02 | .880 | < .001 |
| Face (post) | 98.74 (4.08) | 99.05 (4.68) | | 0.10 | .758 | < .001 |
| total | 98.23 (3.40) | 99.13 (2.66) | | 0.14 | .710 | .002 |

*Note*. Mean (*M*), standard deviation (*SD*) for the autism and neurotypical group, *F*-test results (*F*) for parametric testing and effect size (*η*²).

*Table S3.*

One-way gaze data parameters for both distances between individuals.

|  | **Distance** | | |  | | |
| --- | --- | --- | --- | --- | --- | --- |
|  | 131 cm | 151 cm | |  | | |
|  | *M (SD)* | *M (SD)* | | *F*(1, 74) | *p* | *η*² |
| **Total Dwell-time (%)** | |  | |  |  |  |
| Eyes | 25.56 (17.34) | 24.94 (17.27) | | 0.05 | .821 | < .001 |
| Nose | 9.35 (8.02) | 10.00 (9.02) | | 0.11 | .740 | .0002 |
| Mouth | 7.33 (8.08) | 8.90 (9.50) | | 1.64 | .204 | .022 |
| Rest of Face | 10.74 (6.25) | 13.84 (8.07) | | 2.16 | .146 | .029 |
| Face | 51.94 (20.64) | 57.40 (17.52) | | 1.49 | .226 | .020 |
| Background | 5.21 (6.89) | 4.57 (7.60) | | 0.20 | .653 | .003 |
| **Total Fixation Time (%)** | |  | |  |  |  |
| Eyes | 18.85 (15.13) | 18.01 (14.25) | | 0.05 | .827 | < .001 |
| Nose | 6.62 (6.62) | 6.93 (6.93) | | 0.04 | .844 | < .001 |
| Mouth | 4.32 (8.29) | 5.44 (6.02) | | 0.55 | .461 | .008 |
| Rest of Face | 5.54 (4.37) | 7.09 (4.95) | | 1.68 | .199 | .023 |
| Face | 35.32 (18.31) | 37.41 (17.38) | | 0.25 | .617 | .003 |
| Background | 2.11 (2.68) | 2.12 (2.73) | | < 0.01 | .312 | .014 |
| **Mean Fixation Duration (Sec.)** | |  | |  |  |  |
| Eyes | 0.89 (0.44) | 0.92 (0.72) | | 0.35 | .557 | .005 |
| Nose | 0.83 (0.37) | 0.86 (0.53) | | 0.16 | .684 | .002 |
| Mouth | 0.85 (0.60) | 0.87 (0.58) | | 0.64 | .428 | .009 |
| Rest of Face | 0.75 (0.35) | 0.73 (0.36) | | 0.20 | .653 | .003 |
| Face | 0.82 (0.31) | 0.86 (0.42) | | 0.10 | .758 | .001 |
| Background | 0.47 (0.36) | 0.50 (0.33) | | 2.07 | .155 | .028 |
| **Total Number of Fixations (N)** | |  |  |  |  |  |
| Eyes | 343.26 (233.96) | 342.21 (250.72) | | < 0.01 | .988 | < .001 |
| Nose | 261.69 (275.44) | 275.29 (242.70) | | 0.05 | .823 | < .001 |
| Mouth | 163.13 (175.59) | 210.74 (236.86) | | 0.98 | .326 | .013 |
| Rest of Face | 256.76 (212.48) | 337.74 (232.37) | | 2.45 | .121 | .033 |
| Face | 1008.85 (486.69) | 1168.54 (454.88) | | 2.11 | .150 | .029 |
| Background | 136.39 (196.47) | 113.03 (126.12) | | 0.36 | .550 | .005 |

*Note*. Mean (*M*), standard deviation (*SD*) for the autism and neurotypical group, *F*-test results (*F*) for parametric testing and effect size (*η*²).

*Table S4.*

Two-way gaze data parameters for both distances between individuals.

|  | **Distance** | |  | | |
| --- | --- | --- | --- | --- | --- |
|  | 131 cm | 151 cm |  | | |
|  | *M (SD)* | *M (SD)* | *F*(1, 74) | *p* | *η*² |
| **Mutual Eye Gaze** |  |  |  |  |  |
| Total duration (%) | 15.32 (6.83) | 15.15 (6.34) | 0.76 | .606 | .004 |
| Mean duration (Sec.) | 2.48 (2.48) | 2.52 (2.57) | 0.49 | .653 | .003 |
| **Mutual E Gaze** |  |  |  |  |  |
| Total duration (%) | 21.86 (9.77) | 21.61 (8.77) | 0.42 | .766 | .003 |
| Mean duration (Sec.) | 2.49 (2.52) | 2.53 (2.49) | 0.38 | .804 | .001 |
| **Initiations** |  |  |  |  |  |
| Total number (%) | 52.84 (16.54) | 53.02 (15.56) | 0.56 | .646 | .005 |
| Mean duration (Sec.) | 3.17 (0.94) | 3.21 (1.23) | 0.39 | .743 | .004 |
| **Break ups** |  |  |  |  |  |
| Total (%) | 51.02 (18.56) | 50.37 (20.25) | 0.84 | .451 | .081 |
| When initiated (%) | 54.35 (26.36) | 53.54 (27.32) | 0.57 | .763 | .005 |
| When responded (%) | 53.50 (26.42) | 55.62 (27.14) | 0.70 | .232 | .010 |

*Note*. Mean (*M*), standard deviation (*SD*) for the autism and neurotypical group, *F*-test results (*F*) for parametric testing and effect size (*η*²).

*Table S5.*

Fast Friends procedure: Twelve items in German (applied) and in English (original).

| Example: „Wo isst du am liebsten zu Abend?“  (“*Where is your favorite place to have dinner?”*)   1. „Wenn du dich mit irgendeinem Menschen auf der Welt zum Abendessen verabreden dürftest, wen würdest du wählen?“   (“*Given the choice of anyone in the world, whom would you want as a dinner guest?”)*   1. „Wärst du gerne berühmt? Wenn ja wofür?“   *(Would you like to be famous? In what way*?”)   1. „Gehst du manchmal vor einem Telefonat durch, was du sagen möchtest? Warum?”   *(“Before making a call, do you ever rehearse what you are going to say? Why?“)*   1. „Was würde einen perfekten Tag für dich ausmachen?“   *(“What would constitute a “perfect” day for you?”)*   1. „Wann hast du das letzte Mal für dich alleine gesungen? Wann vor jemand anderem?” (“*When did you last sing to yourself? To someone else?”)* 2. „Wenn du in der Lage wärst, entweder den Geist oder den Körper eines 25-Jährigen dein ganzes Leben lang zu bewahren, was würdest du wählen und warum?“ *(“If you were able to live to the age of 90 and retain either the mind or body of a 30-year-old for the last 60 years of your life, which would you want?”)* 3. „Wenn du morgen mit einer neuen Eigenschaft oder Fähigkeit deiner Wahl aufwachen könntest, welche wäre das? Warum?” *(“If you could wake up tomorrow having gained any one quality or ability, what would it be and why?”)* 4. „Für was in deinem Leben bist du am dankbarsten? Warum?"   *(“For what in your life do you feel most grateful? Why?”)*   1. „Wenn du etwas daran ändern könntest, wie du aufgewachsen bist, was wäre das und warum?“ *(“If you could change anything about the way you were raised, what would it be ad why?”)* 2. „Wenn eine Kristallkugel dir die Wahrheit über dich, dein Leben, die Zukunft oder irgendetwas anderes verraten könnte, was würdest du wissen wollen? Warum?»   (“*If a crystal ball could tell you the truth about yourself, your life, the future, or anything else, what would you want to know?”)*   1. “Nimm dir drei Minuten Zeit und erzähle deinem Partner deine Lebensgeschichte.“   (“*Take four minutes and tell your partner your life story in as much detail as possible.”*) |
| --- |

*Note.* Original questions in English are displayed in bracket

*Table S6.*

Subjective Rating Items: Original items and English translation.

| 1. „Ich bin gestresst.“ *(„I am stressed“*) 2. „Ich fühle mich gut.“ („*I feel good“*) 3. „Ich schäme mich.“ („*I am ashamed“*) 4. „Ich habe Angst.“ („*I'm afraid“*) 5. „Ich möchte eine vertraute Person an meiner Seite.“   (“*I want someone I can trust by my side”*)   1. „Ich möchte die Situation verlassen.“   (“*I want to leave the situation”*)   1. „Wie sympathisch finden Sie Ihren Gesprächspartner?“   (“*How likeable do you find your conversation partner?”)*   1. „Wie authentisch fanden Sie die Gesprächssituation?“   (“*How authentic did you find the interview situation?”*)   1. „Wie sehr hat Ihnen das Gespräch gefallen?“   (“*How much did you enjoy the conversation?”*)   1. „Wie attraktiv finden Sie Ihren Gesprächspartner?“   (“*How attractive do you find your conversation partner?”*)   1. „Wie stark waren Sie durch die Umgebung vom Gespräch abgelenkt?“   (“To what extent were you distracted from the conversation by the environment?”)   1. „Wie sehr mögen Sie Ihren Gesprächspartner?“   (“*How much do you like your conversation partner?”*)   1. „Wie sehr hat Ihnen Ihre Rolle im Gespräch gefallen?“   (“How much did you like your role in the interview?”)   1. „Wie viel haben Sie und die andere Person während des Gesprächs gelacht?“   (“*How much did you and the other person laugh during the conversation?”)*   1. „Wie viel Spaß hat das Gespräch insgesamt gemacht?“   (“*Overall, how much fun was the interview?”)*   1. „Wie viele persönliche oder vertrauliche Informationen hat die andere Person mit Ihnen geteilt?“   (“*How much personal or confidential information did the other person share with you?”)*   1. „Ich würde in einem nächsten Experiment wieder gerne mit diesem Gesprächspartner zusammenarbeiten.“   (“*I would like to work with this interlocutor again in a future experiment”*)   1. „Wie offen und ehrlich war Ihr Gesprächspartner?“   (“*How open and honest was your conversation partner?”*)   1. „Wie offen und ehrlich waren Sie selbst?“   (“*How open and honest were you yourself?”*) |
| --- |

*Note.* Items 1 – 6 were presented pre and post the interaction, Items 7-19 only post the interaction. The translation into English is given in brackets.

*Table S7.*

Mean mutual face gaze duration for both groups with varying thresholds for event length.

|  | **Autism** | **Neurotypical** |  | | |
| --- | --- | --- | --- | --- | --- |
| Threshold | *M (SD)* | *M (SD)* | *F*(1, 74) | *p* | *η*² |
| 0 ms | 12.88 (9.47) | 17.82 (10.83) | 10.67 | < .001 | .073 |
| 100 ms | 12.64 (9.03) | 17.54 (10.39) | 11.32 | < .001 | .084 |
| 200 ms | 12.64 (10.08) | 17.45 (10.47) | 7.01 | .003 | .016 |
| 250 ms | 12.22 (9.92) | 17.53 (10.23) | 10.22 | < .001 | .090 |
| 300 ms | 11.09 19.97) | 17.40 (10.27) | 11.19 | < .001 | .086 |
| 500 ms | 10.88 (9.90) | 16.42 (10.52) | 09.33 | < .001 | .079 |
| 750 ms | 9.17 (9.96) | 16.46 (10.75) | 10.10 | < .001 | .075 |
| 1000 ms | 7.38 (10.08) | 15.49 (11.24) | 09.73 | < .001 | .074 |

*Note.* Events were classified for analysis of two-way gaze behavior. Effect of group was not impacted by variation of threshold for minimum length. The distribution shows that there was no difference in these events between the two groups and thus no systematic error occurred. Mean (*M*), standard deviation (*SD*) for the autism and neurotypical group, *F*-test results (*F*) for parametric testing and effect size (*η*²).

*Table S8.*

One-way gaze data of confederates interacting with the autism and neurotypical group

|  | Confederates: Autism | Confederates: Neurotypical | |  | | |  | |
| --- | --- | --- | --- | --- | --- | --- | --- | --- |
|  | *M (SD)* | *M (SD)* | | *F*(1, 74) | *p* | η² | *Z* | *p* |
| **Total Dwell-time (%)** | | | |  |  |  |  |  |
| Eyes | 27.78 (10.07) | 30.20 (6.98) | | 1.43 | .235 | .020 | - 0.91 | .361 |
| Nose | 9.49 (9.03) | 7.48 (7.06) | | 1.14 | .290 | .016 | - 0.64 | .520 |
| Mouth | 2.23 (2.73) | 2.05 (1.82) | | 0.11 | .737 | .002 | - 0.22 | .825 |
| Rest of Face | 12.50 (6.00) | 9.33 (5.67) | | 5.48 | .110* | .071 | - 2.85 | .020* |
| Face | 52.00 (11.64) | 49.05 (9.58) | | 1.42 | .237 | .019 | - 1.20 | 232 |
| Background | 3.87 (2.84) | 5.39 (3.56) | | 4.08 | .235* | .054 | - 2.09 | .180* |
| **Total Fixation Time (%)** | | | |  |  |  |  |  |
| Eyes | 21.78 (8.79) | 22.95 (5.43) | | 0.47 | .495 | .007 | - 0.37 | .709 |
| Nose | 7.22 (8.09) | 5.39 (6.38) | | 1.17 | .284 | .016 | - 0.76 | .446 |
| Mouth | 1.44 (2.32) | 1.13 (1.36) | | 0.50 | .481 | .007 | - 0.78 | .433 |
| Rest of Face | 7.74 (4.67) | 5.18 (3.98) | | 6.45 | .065* | .082 | - 2.95 | .015* |
| Face | 38.18 (11.17) | 34.64 (8.36) | | 2.38 | .127 | .032 | - 1.83 | .067 |
| Background | 2.29 (1.92) | 3.23 (2.13) | | 3.90 | .110* | .051 | - 2.33 | .100* |
| **Mean Fixation Duration (Sec.)** | | | |  |  |  |  |  |
| Eyes | 1.16 (0.32) | 1.08 (0.33) | | 0.94 | .336 | .013 | - 1.76 | .079 |
| Nose | 0.91 (0.54) | 0.86 (0.55) | | 0.13 | .723 | .002 | - 0.49 | .623 |
| Mouth | 0.53 (0.43) | 0.48 (0.36) | | 0.20 | .660 | .003 | - 0.18 | .858 |
| Rest of Face | 0.88 (0.28) | 0.77 (0.26) | | 2.92 | .092 | .039 | - 1.87 | .062 |
| Face | 0.87 (0.29) | 0.80 (0.23) | | 1.23 | .271 | .017 | - 0.87 | .284 |
| Background | 0.98 (0.63) | 1.01 (0.65) | | 0.04 | .838 | .001 | - 0.20 | .841 |
| **Total Number of Fixations (N)** | | |  |  |  |  |  |  |
| Eyes | 742.22 (315.08) | 842.00 (286.02) | | 2.03 | .158 | .027 | - 1.24 | .216 |
| Nose | 224.16 (178.99) | 171.92 (128.18) | | 2.08 | .153 | .028 | - 0.84 | .402 |
| Mouth | 48.65 (63.47) | 43.65 (42.18) | | 0.16 | .691 | .002 | - 0.45 | .650 |
| Rest of Face | 331.57 (175.36) | 241.24 (156.30) | | 5.47 | .110* | .071 | - 2.66 | .040* |
| Face | 1346.60 (732.90) | 1298.81(612.68) | | 0.37 | .547 | .005 | - 1.22 | .224 |
| Background | 83.30 (77.14) | 122.70 (100.26) | | 3.59 | .062 | .047 | - 2.02 | .215* |

*Note*.; *M*: Mean; *SD*: Standard Deviation; *F* = F-values with degrees of freedom, *p* = p-values, η² = partial eta square; *Bonferroni corrected

*Table S9.*

Subjective rating items for confederates interacting with both groups.

|  | Confederate:  Autism | | Confederate:  Neurotypical | |  | | |
| --- | --- | --- | --- | --- | --- | --- | --- |
| **Item** | *M (SD)* | | *M (SD)* | | *F*(1, 74) | *p* | *η*² |
| Shame (pre) | 6.14 (8.11) | | 5.43 (6.17) | | 0.18 | .676 | .002 |
| Fear (pre) | 4.86 (6.41) | | 5.16 (4.98) | | 0.05 | .824 | .001 |
| Happy (pre) | 77.22 (12.37) | | 77.86 (12.22) | | 0.05 | .821 | .001 |
| Wish to leave (pre) | 10.57 (15.96) | | 9.76 (12.60) | | 0.06 | .809 | .001 |
| Trustee (pre) | 15.97 (24.65) | | 10.78 (16.01) | | 1.15 | .286 | .016 |
| Stress (pre) | 12.30 (13.92) | | 14.51 (19.65) | | 0.31 | .577 | .004 |
| Shame (post) | 5.32 (9.51) | | 3.70 (4.58) | | 0.87 | .353 | .012 |
| Fear (post) | 5.32 (8.86) | | 4.46 (5.67) | | 0.25 | .619 | .003 |
| Happy (post) | 79.27 (12.41) | | 81.73 (11.42) | | 0.79 | .378 | .011 |
| Wish to leave (post) | 10.49 (15.26) | | 10.24 (13.84) | | 0.01 | .943 | .000 |
| Trustee (post) | 13.35 (20.49) | | 10.30 (16.21) | | 0.51 | .479 | .007 |
| Stress (post) | 9.92 (11.82) | | 11.35 (16.88) | | 0.18 | .674 | .002 |
|  |  |  |  |  |  |  |  |
| Sympathy | 72.78 (20.30) | | 71.51 (16.60) | | 0.09 | .769 | .001 |
| Authenticity | 68.19 (20.91) | | 58.57 (21.27) | | 3.85 | .054 | .051 |
| Attractiveness | 48.41 (24.69) | | 49.51 (21.89) | | 0.04 | .839 | .001 |
| Liking | 72.86 (18.21) | | 67.65 (14.53) | | 1.86 | .177 | .025 |
| Distraction | 6.32 (7.25) | | 5.43 (6.97) | | 0.29 | .591 | .004 |
| Enjoyment | 76.54 (17.87) | | 69.78 (17.21) | | 2.74 | .102 | .037 |
| Enjoyment of role | 74.59 (16.51) | | 63.62 (21.31) | | 6.13 | .016* | .079 |
| Laughing | 54.65 (30.27) | | 57.57 (19.44) | | 0.24 | .623 | .003 |
| Fun | 77.03 (15.82) | | 71.41 (16.64) | | 2.22 | .141 | .030 |
| Revealed details(self) | 75.22 (14.47) | | 71.68 (12.34) | | 1.28 | .261 | .018 |
| Revealed details (opponent) | 71.84 (21.67) | | 73.54 (21.81) | | 0.11 | .737 | .002 |
| Openness (opponent) | 75.43 (15.16) | | 73.24 (13.75) | | 0.42 | .517 | .006 |
| Openness (own) | 68.08 (25.42) | | 53.86 (24.10) | | 6.09 | .016* | 078 |
|  |  |  |  |  |  |  |  |
| IOS (actual) | 4.38 (1.53) | | 4.11 (1.39) | | 0.63 | .430 | .009 |
| IOS (desired) | 4.62 (1.57) | | 4.08 (1.30) | | 2.60 | .111 | .035 |

*Note.* Subjective Ratings for both groups Items 1 – 6 were presented twice (pre and post interaction), Items 7-19 only after the interaction; IOS: Inclusion of Other in the Self Scale; *M*: Mean; *SD*: Standard Deviation; *F* = F-values with degrees of freedom; *p* = p-values; η² = partial eta square; *** *p* < 0.05, *** p* < .01, **** p* < .001

*Table S10.*

One-way gaze data parameters for autism and neurotypical group.

|  | **Autism** | **Neurotypical** | |  | | |
| --- | --- | --- | --- | --- | --- | --- |
|  | *M (SD)* | *M (SD)* | | *F*(1, 74) | *p* | *η*² |
| **Total Dwell-time (%)** | |  | |  |  |  |
| Eyes | 18.77 (15.48) | 31.48 (16.64) | | 10.29 | .002 | .125 |
| Nose | 7.38 (6.86) | 11.92 (9.34) | | 5.73 | .019 | .074 |
| Mouth | 7.01 (10.18) | 8.07 (7.31) | | 0.72 | .299 | .010 |
| Rest of Face | 12.21 (10.82) | 12.20 (7.21) | | 0.31 | .578 | .004 |
| Face | 45.37 (20.83) | 63.67 (12.18) | | 23.91 | <.001 | .249 |
| Background | 6.61 (7.93) | 3.23 (1.95) | | 7.91 | .006 | .099 |
| **Total Fixation Time (%)** | |  | |  |  |  |
| Eyes | 12.81 (11.68) | 24.17 (15.19) | | 11.48 | .001 | .137 |
| Nose | 5.02 (5.21) | 8.52 (7.62) | | 5.49 | .022 | .071 |
| Mouth | 3.86 (6.98) | 5.84 (5.93) | | 2.74 | .103 | .037 |
| Rest of Face | 5.70 (5.15) | 6.69 (4.17) | | 0.75 | .390 | .010 |
| Face | 27.40 (16.73) | 45.25 (14.08) | | 24.26 | <.001 | .252 |
| Background | 2.90 (3.54) | 1.32 (0.90) | | 8.75 | .004 | .108 |
| **Mean Fixation Duration (Sec.)** | |  | |  |  |  |
| Eyes | 0.69 (0.43) | 1.09 (0.66) | | 10.77 | .002 | .130 |
| Nose | 0.77 (0.43) | 0.92 (0.46) | | 2.08 | .153 | .028 |
| Mouth | 0.75 (0.63) | 0.88 (0.47) | | 1.49 | .226 | .020 |
| Rest of Face | 0.67 (0.36) | 0.81 (0.34) | | 2.18 | .144 | .029 |
| Face | 0.72 (0.35) | 0.93 (0.35) | | 7.00 | .010 | .089 |
| Background | 0.55 (0.46) | 0.41 (0.15) | | 3.94 | .051 | .052 |
| **Total Number of Fixations (N)** | |  |  |  |  |  |
| Eyes | 577.41 (500.49) | 812.62 (420.11) | | 4.79 | .032 | 0.62 |
| Nose | 219.14 (266.06) | 326.68 (249.25) | | 3.22 | .077 | 0.43 |
| Mouth | 147.70 (188.31) | 223.59 (219.71) | | 2.55 | .115 | .034 |
| Rest of Face | 265.49 (221.60) | 290.92 (192.82) | | 0.27 | .600 | .004 |
| Face | 1209.73 (683.54) | 1653.81 (454.95) | | 10.82 | .002 | .131 |
| Background | 173.73 (220.12) | 75.41 (49.04) | | 7.03 | .010 | .089 |

*Note*. Mean (*M*), standard deviation (*SD*) for the autism and neurotypical group, *F*-test results (*F*) for parametric testing and effect size (*η*²).

*Table S11.*

Generalized linear mixed model indices for variables total dwell-time on AOIs.

| Model:  dependent variable | Random Slopes | *SD* | Fixed Effects | *b*(SDE) | *t*(74) | *p* | Δ AIC |
| --- | --- | --- | --- | --- | --- | --- | --- |
| **Model 1:**  Total dwell-time on face (%) | Question | 0.42 | Group | 0.89 (0.17) | 5.18 | < .001 | 26.08 |
|  | Phase | 0.33 | Phase | 0.26 (0.07) | 3.97 | < .001 | 12.05 |
|  |  |  | Time | 0.22 (0.06) | 0.32 | .426 | 1.24 |
|  |  |  | Group*Phase | 0.11 (0.04) | 0.90 | .367 | 0.93 |
|  |  |  |  |  |  |  |  |
| **Model 2:**  Total dwell-time on eyes (%) | Question | 0.41 | Group | 0.68 (0.18) | 11.40 | < .001 | 10.59 |
|  | Phase | 0.32 | Phase | 0.04 (0.09) | 21.27 | .006 | 5.26 |
|  |  |  | Time | 0.12 (0.10) | 0.33 | .473 | 1.13 |
|  |  |  | Group*Phase | 0.10 (0.12) | 23.66 | .009 | 3.63 |
|  |  |  |  |  |  |  |  |
| **Model 3:**  Total dwell-time on nose (%) | Question | 0.34 | Group | 0.67 (0.20) | 3.86 | < .001 | 11.01 |
|  | Phase | 0.24 | Phase | 0.10 (0.08) | 1.04 | .331 | 0.49 |
|  |  |  | Time | 0.14 (0.04) | 0.42 | .437 | 0.47 |
|  |  |  |  |  |  |  |  |
| **Model 4:**  Total dwell-time on mouth (%) | Question | 0.32 | Group | 0.64 (0.17) | 3.31 | < .001 | 9.37 |
|  | Phase | 0.29 | Phase | 0.08 (0.09) | 1.51 | .095 | 1.49 |
|  |  |  | Time | 0.52 (0.09) | .044 | .351 | 0.71 |
|  |  |  |  |  |  |  |  |
| **Model 5:**  Total fixation time rest of face (%) | Question | 0.35 | Group | 0.31 (0.16) | 1.13 | .134 | 1.91 |
|  | Phase | 0.25 | Phase | 0.10 (0.08) | 0.95 | .189 | 0.83 |
|  |  |  | Time | 0.10 (0.04) | 0.35 | .453 | 0.76 |
|  |  |  |  |  |  |  |  |
| **Model 6:**  Total dwell-time on background (%) | Question | 0.32 | Group | 0.81 (0.18) | 4.64 | < .001 | 11.03 |
|  | Phase | 0.24 | Phase | 0.27 (0.08) | 3.92 | < .001 | 9.52 |
|  |  |  | Time | 0.31 (0.09) | 0.44 | .456 | 0.46 |
|  |  |  | Group*Phase | 0.09 (0.06) | 0.87 | .217 | 0.67 |

*Note.* Regression coefficients (b), standard errors (SE), *t*-test results with degrees of freedom (*t*), p-values (*p*), Akaike Information Criterion difference (Δ AIC)

*Table S12.*

Generalized linear mixed model indices for variables total fixation time on AOIs.

| Model:  dependent variable | Random Slopes | *SD* | Fixed Effects | *b*(SDE) | *t*(74) | *p* | Δ AIC |
| --- | --- | --- | --- | --- | --- | --- | --- |
| **Model 1:**  Total fixation time on face (%) | Question | 0.42 | Group | 0.83 (0.18) | 5.04 | < .001 | 16.11 |
|  | Phase | 0.34 | Phase | 0.25 (0.08) | 3.81 | < .001 | 13.85 |
|  |  |  | Time | 0.22 (0.07) | 0.32 | .426 | 0.28 |
|  |  |  | Group*Phase | 0.27 (0.07) | 1.04 | .243 | 0.37 |
|  |  |  |  |  |  |  |  |
| **Model 2:**  Total fixation time on eyes (%) | Question | 0.41 | Group | 0.68 (0.18) | 4.98 | < .001 | 10.83 |
|  | Phase | 0.32 | Phase | 0.03 (0.07) | 3.34 | .682 | 1.45 |
|  |  |  | Time | 0.13 (0.03) | 0.39 | .423 | 0.42 |
|  |  |  |  |  |  |  |  |
| **Model 3:**  Total fixation time on nose (%) | Question | 0.32 | Group | 0.69 (0.18) | 3.76 | < .001 | 10.73 |
|  | Phase | 0.27 | Phase | 0.09 (0.08) | 1.01 | .313 | 0.53 |
|  |  |  | Time | 0.13 (0.03) | 0.39 | .423 | 0.42 |
|  |  |  |  |  |  |  |  |
| **Model 4:**  Total fixation time on mouth (%) | Question | 0.37 | Group | 0.62 (0.18) | 3.76 | < .001 | 9.24 |
|  | Phase | 0.29 | Phase | 0.08 (0.09) | 1.51 | .059 | 1.53 |
|  |  |  | Time | 0.12 (0.04) | .051 | .345 | 0.45 |
|  |  |  |  |  |  |  |  |
| **Model 5:**  Total fixation time rest of face (%) | Question | 0.41 | Group | 0.31 (0.16) | 1.13 | .134 | 1.91 |
|  | Phase | 0.30 | Phase | 0.10 (0.08) | 0.95 | .189 | 0.83 |
|  |  |  | Time | 0.10 (0.04) | 0.35 | .453 | 0.76 |
|  |  |  |  |  |  |  |  |
| **Model 6:**  Total fixation time on background (%) | Question | 0.36 | Group | 0.79 (0.19) | 4.54 | < .001 | 11.11 |
|  | Phase | 0.31 | Phase | 0.25 (0.08) | 3.81 | < .001 | 9.52 |
|  |  |  | Time | 0.32 (0.09) | 0.42 | .456 | 0.46 |
|  |  |  | Group*Phase | 0.22 (0.11) | 0.94 | .213 | 0.63 |

*Note.* Regression coefficients (b), standard errors (SE), *t*-test results with degrees of freedom (*t*), p-values (*p*), Akaike Information Criterion difference (Δ AIC)

*Table S13.*

Generalized linear mixed model indices for variables mean fixation duration on AOIs.

| Model:  dependent variable | Random Slopes | *SD* | Fixed Effects | *b*(SDE) | *t*(74) | | *p* | | Δ AIC | |
| --- | --- | --- | --- | --- | --- | --- | --- | --- | --- | --- |
| **Model 1:**  Mean fixation duration on face (Sec) | Question | 0.40 | Group | 0.54 (0.18) | | 5.04 | < .001 | 8.11 | | |
|  | Phase | 0.31 | Phase | 0.75 (0.08) | | 1.71 | .153 | 1.85 | | |
|  |  |  | Time | 0.22 (0.07) | | 0.32 | .426 | 0.28 | | |
|  |  |  |  |  | |  |  |  | | |
| **Model 2:**  Mean fixation duration on eyes (Sec) | Question | 0.45 | Group | 0.68 (0.18) | | 9.04 | < .001 | 1.91 | | |
|  | Phase | 0.34 | Phase | 0.03 (0.07) | | 3.34 | .682 | 1.36 | | |
|  |  |  | Time | 0.13 (0.03) | | 0.39 | .423 | 0.45 | | |
|  |  |  |  |  | |  |  |  | | |
| **Model 3:**  Mean fixation duration on nose (Sec) | Question | 0.31 | Group | 0.20 (0.11) | | 0.56 | .492 | 1.03 | | |
|  | Phase | 0.25 | Phase | 0.09 (0.04) | | 0.71 | .313 | 0.73 | | |
|  |  |  | Time | 0.13 (0.04) | | 0.57 | .423 | 0.62 | | |
|  |  |  |  |  | |  |  |  | | |
| **Model 4:**  Mean fixation duration on mouth (Sec) | Question | 0.35 | Group | 0.10 (0.07) | | 0.53 | .473 | 1.34 | | |
|  | Phase | 0.28 | Phase | 0.09 (0.09) | | 0.63 | .392 | 1.03 | | |
|  |  |  | Time | 0.11 (0.10) | | 0.51 | .641 | 0.45 | | |
|  |  |  |  |  | |  |  |  | | |
| **Model 5:**  Total fixation time rest of face (Sec) | Question | 0.42 | Group | 0.05 (0.05) | | 0.53 | .628 | 1.35 | | |
|  | Phase | 0.31 | Phase | 0.04 (0.05) | | 0.42 | .572 | 0.63 | | |
|  |  |  | Time | 0.02 (0.04) | | 0.42 | .462 | 0.53 | | |
|  |  |  |  |  | |  |  |  | | |
| **Model 6:**  Total fixation time on background (%Sec) | Question | 0.32 | Group | 0.10 (0.07) | | 0.43 | .324 | 0.89 | | |
|  | Phase | 0.20 | Phase | 0.09 (0.06) | | 0.54 | .527 | 0.55 | | |
|  |  |  | Time | 0.04 (0.03) | | 0.42 | .754 | 0.46 | | |
|  |  |  |  |  | |  |  | | |  |

*Note.* Regression coefficients (b), standard errors (SE), *t*-test results with degrees of freedom (*t*), p-values (*p*), Akaike Information Criterion difference (Δ AIC)

*Table S14.*

Generalized linear mixed model indices for variables number of fixations on AOIs.

| Model:  Dependent variable | Random Slopes | *SD* | Fixed Effects | *b*(SDE) | *t*(74) | | *p* | | Δ AIC |
| --- | --- | --- | --- | --- | --- | --- | --- | --- | --- |
| **Model 1:**  Mean fixation duration on face (Sec) | Question | 0.40 | Group | 0.63 (0.17) | | 5.16 | < .001 | 7.43 | |
|  | Phase | 0.31 | Phase | 0.54 (0.12) | | 1.58 | .186 | 1.92 | |
|  |  |  | Time | 0.22 (0.07) | | 0.43 | .464 | 0.31 | |
|  |  |  |  |  | |  |  |  | |
| **Model 2:**  Mean fixation duration on eyes (Sec) | Question | 0.42 | Group | 0.73 (0.22) | | 7.30 | < .001 | 2.83 | |
|  | Phase | 0.30 | Phase | 0.10 (0.03) | | 3.34 | .582 | 1.32 | |
|  |  |  | Time | 0.13 (0.03) | | 0.39 | .387 | 0.62 | |
|  |  |  |  |  | |  |  |  | |
| **Model 3:**  Mean fixation duration on nose (Sec) | Question | 0.40 | Group | 0.17 (0.10) | | 1.36 | .240 | 1.45 | |
|  | Phase | 0.33 | Phase | 0.13 (0.07) | | 0.81 | .532 | 0.31 | |
|  |  |  | Time | 0.05 (0.03) | | 0.45 | .352 | 0.63 | |
|  |  |  |  |  | |  |  |  | |
| **Model 4:**  Mean fixation duration on mouth (Sec) | Question | 0.36 | Group | 0.34 (0.13) | | 0.84 | .253 | 1.64 | |
|  | Phase | 0.22 | Phase | 0.20 (0.07) | | 0.50 | .193 | 1.73 | |
|  |  |  | Time | 0.12 (0.06) | | 0.38 | .244 | 0.33 | |
|  |  |  |  |  | |  |  |  | |
| **Model 5:**  Total fixation time rest of face (Sec) | Question | 0.39 | Group | 0.14 (0.10) | | 1.13 | .630 | 1.46 | |
|  | Phase | 0.31 | Phase | 0.25 (0.15) | | 0.95 | .329 | 0.56 | |
|  |  |  | Time | 0.12 (0.07) | | 0.35 | .453 | 0.43 | |
|  |  |  |  |  | |  |  |  | |
| **Model 6:**  Total fixation time on background (%Sec) | Question | 0.31 | Group | 0.32 (0.06) | | 5.14 | < .001 | 6.42 | |
|  | Phase | 0.29 | Phase | 0.17 (0.09) | | 1.75 | < .001 | 2.32 | |
|  |  |  | Time | 0.26 (0.10) | | 0.57 | .283 | 0.46 | |
|  |  |  |  |  | |  |  | |  |

*Note.* Regression coefficients (b), standard errors (SE), *t*-test results with degrees of freedom (*t*), p-values (*p*), Akaike Information Criterion difference (Δ AIC)

*Table S15.*

Two-way gaze data variables for the autism and neurotypical group.

|  | **Autism** | **Neurotypical** |  | | |
| --- | --- | --- | --- | --- | --- |
|  | *M (SD)* | *M (SD)* | *F*(1, 74) | *p* | *η*² |
| **Mutual Eye Gaze** |  |  |  |  |  |
| Total duration (%) | 12.65 (7.08) | 17.42 (5.47) | 10.51 | .002 | .127 |
| Mean duration (Sec.) | 1.97 (1.23) | 3.03 (1.91) | 7.99 | .006 | .101 |
| **Mutual Face Gaze** |  |  |  |  |  |
| Total duration (%) | 18.03 (10.10) | 24.85 (7.51) | 10.85 | .002 | .131 |
| Mean duration (Sec.) | 1.69 (1.74) | 3.11 (2.27) | 13.08 | <.001 | .019 |
| **Initiations** |  |  |  |  |  |
| Total number (%) | 44.16 (15.87) | 61.99 (10.45) | 15.82 | <.001 | .182 |
| Mean duration (Sec.) | 3.05 (1.39) | 3.92 (1.83) | 4.75 | .003 | .063 |
| **Break ups** |  |  |  |  |  |
| Total (%) | 62.97 (19.61) | 41.09 (14.93) | 29.15 | <.001 | .288 |
| When initiated (%) | 60.31 (26.26) | 38.24 (27.57) | 23.92 | <.001 | .177 |
| When responded (%) | 64.56 (26.35) | 45.52 (27.89) | 20.18 | <.001 | .141 |

*Note*. Mean (*M*), standard deviation (*SD*) for the autism and neurotypical group, *F*-test results (*F*) for parametric testing and effect size (*η*²).

*Table S16.*

Generalized linear mixed model indices for two-way gaze data

| Model:  dependent variable | Random Slopes | *SD* | Fixed Effect | *b*(SDE) | *t*(74) | | *p* | Δ AIC | |
| --- | --- | --- | --- | --- | --- | --- | --- | --- | --- |
| **Model 1:**  Total mutual eye gaze duration (%) | Question | 0.49 | Group | 0.31 (0.05) | | 56.16 | < .001 | | 95.17 |
|  | Phase | 0.36 | Phase | 0.36 (0.08) | | 13.11 | < .001 | | 54.35 |
|  |  |  | Time | 0.05 (0.08) | | 0.35 | .634 | | 5.94 |
|  |  |  | Group*Phase | 0.26 (0.06) | | 9.15 | < .001 | | 9.31 |
|  |  |  |  |  | |  |  | |  |
| **Model 2:**  Total mutual face gaze duration (%) | Question | 0.53 | Group | 0.35 (0.11) | | 54.24 | < .001 | | 104.94 |
|  | Phase | 0.34 | Phase | 0.40 (0.08) | | 14.05 | < .001 | | 85.93 |
|  |  |  | Time | 0.04 (0.09) | | 0.39 | .617 | | 5.21 |
|  |  |  | Group*Phase | 0.47 (0.08) | | 14.05 | < .001 | | 31.34 |
|  |  |  |  |  | |  |  | |  |
| **Model 3:**  Initiations (%) | Question | 0.38 | Group | 0.34 (0.10) | | 46.16 | < .001 | | 64.75 |
|  | Phase | 0.29 | Phase | 0.32 (0.09) | | 13.11 | < .001 | | 41.30 |
|  |  |  | Time | 0.02 (0.05) | | 0.29 | .689 | | 4.50 |
|  |  |  | Group*Phase | 0.07 (0.05) | | 0.41 | .409 | | 3.36 |
|  |  |  |  |  | |  |  | |  |
| **Model 4:**  Break-ups (%) | Question | 0.36 | Group | 0.25 (0.13) | | 54.24 | < .001 | | 90.03 |
|  | Phase | 0.30 | Phase | 0.34 (0.08) | | 12.47 | < .001 | | 85.93 |
|  |  |  | Time | 0.03 (0.09) | | 0.54 | .591 | | 3.13 |
|  |  |  | Group*Phase | 0.39 (0.09) | | 7.21 | < .001 | | 19.75 |

*Note.* Regression coefficients (b), standard errors (SE), *t*-test results with degrees of freedom (*t*), p-values (*p*), Akaike Information Criterion difference (Δ AIC)

*Table S17.*

Subjective rating items for both groups.

| **Item** | **Autism** | | **Neurotypical** | |  | | |
| --- | --- | --- | --- | --- | --- | --- | --- |
|  | *M (SD)* | | *M* *(SD)* | | *F*(1, 74) | *p* | *η*² |
| Shame (pre) | 17.26 (24.08) | | 7.49 (9.90) | | 5.42* | .022 | .070 |
| Fear (pre) | 21.35 (22.34) | | 13.73 (19.85) | | 2.41* | .033 | .032 |
| Happy (pre) | 61.76 (24.56) | | 74.62 (22.89) | | 5.43* | .023 | .070 |
| Wish to leave (pre) | 23.86 (27.02) | | 14.76 (17.01) | | 3.09 | .083 | .041 |
| Trustee (pre) | 36.49 (34.51) | | 30.22 (26.14) | | 0.77 | .381 | .011 |
| Stress (pre) | 36.22 (27.30) | | 23.19 (20.20) | | 5.44* | .022 | .070 |
| Shame (post) | 13.05 (20.97) | | 6.43 (9.30) | | 3.08 | .083 | .041 |
| Fear (post) | 12.05 (18.11) | | 5.68 (7.25) | | 3.96* | .051 | .052 |
| Happy (post) | 69.89 (19.84) | | 82.65 (15.61) | | 9.45** | < .003 | .116 |
| Wish to leave (post) | 16.81 (22.49) | | 9.57 (14.30) | | 2.73 | .103 | .037 |
| Trustee (post) | 19.62 (23.24) | | 14.92 (19.24) | | 0.90 | .346 | .012 |
| Stress (post) | 22.78 (22.12) | | 11.81 (14.57) | | 6.35* | .014 | .081 |
|  |  |  |  |  |  |  |  |
| Sympathy | 71.16 (13.48) | | 76.87 (15.78) | | 2.90 | .093 | .039 |
| Authenticity | 50.76 (27.29) | | 56.24 (25.92) | | 0.78 | .378 | .011 |
| Attractiveness | 44.19 (23.72) | | 39.32 (25.04) | | 0.74 | .394 | .010 |
| Liking | 62.43 (14.73) | | 67.51 (14.59) | | 2.22 | .140 | .030 |
| Distraction | 28.49 (25.35) | | 19.43 (19.16) | | 3.01 | .087 | .040 |
| Enjoyment | 63.30 (21.30) | | 72.19 (13.91) | | 4.60* | .035 | .060 |
| Enjoyment of role | 56.05 (24.03) | | 67.19 (17.27) | | 5.24* | .025 | .068 |
| Laughing | 40.16 (23.10) | | 46.70 (22.39) | | 1.53 | .220 | .021 |
| Fun | 63.00 (20.68) | | 70.86 (14.21) | | 3.64 | .061 | .048 |
| Revealed details (self) | 69.73 (21.11) | | 77.22 (14.06) | | 3.22 | .077 | .043 |
| Revealed details (opponent) | 63.65 (17.81) | | 62.97 (16.41) | | 0.03 | .866 | < .001 |
| Openness (opponent) | 76.08 (16.93) | | 78.95 (16.39) | | 0.55 | .462 | .008 |
| Openness (own) | 83.35 (18.15) | | 85.41 (17.08) | | 0.25 | .618 | .003 |
|  |  |  |  |  |  |  |  |
| IOS (actual) | 2.95 (1.73) | | 3.16 (1.32) | | 0.36 | .548 | .005 |
| IOS (desired) | 2.73 (1.77) | | 3.00 (1.37) | | 0.54 | .466 | .007 |

*Note.* Subjective Ratings for both groups Items 1 – 6 were presented twice (pre and post interaction), Items 7-19 only after the interaction; IOS: Inclusion of Other in the Self Scale; *M*: Mean; *SD*: Standard Deviation; *F* = F-values with degrees of freedom, *p* = p-values, η² = partial eta square; *** *p* < 0.05, *** p* < .01, **** p* < .001

*Table S18.*

Association between subjective ratings and gaze behavior parameters.

|  | Dwell Time on Eyes | | | | | Mutual Eye Gaze | |  |
| --- | --- | --- | --- | --- | --- | --- | --- | --- |
|  |  |  | *r* | *p* | *r* | | *p* | |
| Shame (pre) |  |  | -.249 | .138 | -.094 | | .580 | |
| Fear (pre) |  |  | -.023 | .895 | .168 | | .319 | |
| Happy (pre) |  |  | -.018 | .915 | .194 | | .250 | |
| Wish to leave (pre) |  |  | -.107 | .530 | -.091 | | .592 | |
| Trustee (pre) |  |  | -.073 | .667 | .054 | | .752 | |
| Stress (pre) |  |  | -.189 | .262 | -.086 | | .614 | |
| Shame (post) |  |  | -.277 | .097 | -.397 | | .015 | |
| Fear (post) |  |  | -.227 | .178 | -.356 | | .030* | |
| Happy (post) |  |  | .012 | .944 | .194 | | .251 | |
| Wish to leave (post) |  |  | -.180 | .287 | -.291 | | .081 | |
| Trustee (post) |  |  | .118 | .488 | .075 | | .658 | |
| Stress (post) |  |  | -.200 | .236 | -.373 | | .023* | |
| Sympathy |  |  | .215 | .202 | .504 | | .001* | |
| Authenticity |  |  | -.112 | .508 | .033 | | .845 | |
| Attractiveness |  |  | -.221 | .188 | -.206 | | .221 | |
| Liking |  |  | -.014 | .934 | .064 | | .709 | |
| Distraction |  |  | -.165 | .331 | -.150 | | .376 | |
| Enjoyment |  |  | .206 | .221 | .270 | | .107 | |
| Enjoyment of role |  |  | .115 | .499 | .253 | | .131 | |
| Laughing |  |  | .002 | .999 | .102 | | .547 | |
| Fun |  |  | .216 | .199 | .434 | | .007* | |
| Revealed details (self) |  |  | -.070 | .681 | .042 | | .804 | |
| Revealed details (opponent) |  |  | .291 | .081 | .279 | | .094 | |
| Openness (opponent) |  |  | -.104 | .540 | .006 | | .970 | |
| Openness (own) |  |  | .129 | .446 | .010 | | .955 | |
| IOS (actual) |  |  | -.209 | .215 | -.124 | | .465 | |
| IOS (desired) |  |  | -.199 | .237 | -.126 | | .456 | |

*Note.* Pearson coefficients and p-values for Association between subjective ratings and gaze behavior parameters. *Bonferroni corrected

*Table S19.*

Gaze behavior parameters for Autism low, Autism high, and control group.

|  | Autism low | Autism high | Neurotypical |
| --- | --- | --- | --- |
|  | *M (SD)* | *M (SD)* | *M (SD)* |
| **Total Dwell-time (%)** |  |  |  |
| Eyes | 7.84 *(*7.19) ^a, b^ | 33.83 (9.34) ^a^ | 31.48 (16.64) ^b^ |
| Nose | 7.17 (7.74) ^a, b^ | 8.13 (5.56) ^a^ | 11.92 (9.34) ^b^ |
| Mouth | 9.99 (12.99) | 3.80 (2.92) | 8.07 (7.31) |
| Rest of Face | 14.88 (12.46) | 9.43 (7.45) | 12.20 (7.21) |
| Face | 39.87 (22.18) ^a, b^ | 55.19 (11.68) ^a^ | 63.67 (12.18) ^b^ |
| Background | 9.32 (9.95) ^a, b^ | 3.80 (1.98) ^a^ | 3.23 (1.95) ^b^ |
| **Total Fixation Dur. (%)** |  |  |  |
| Eyes | 4.92 (5.84) ^a, b^ | 5.48 (4.36) ^a^ | 24.17 (15.19) ^b^ |
| Nose | 5.31 (9.10) | 2.35 (2.30) | 8.52 (7.62) |
| Mouth | 6.89 (5.53) | 4.52 (4.26) | 5.84 (5.93) |
| Rest of Face | 4.10 (4.39) ^a, b^ | 1.67 (1.15) ^a^ | 6.69 (4.17) ^b^ |
| Face | 21.81 (17.35) ^a, b^ | 36.52 (10.70) ^a^ | 45.25 (14.08) ^b^ |
| Background | 4.92 (5.84) ^a, b^ | 5.48 (4.36) ^a^ | 1.32 (0.90) ^b^ |
| **Fix. Duration (*M* in Sec.)** |  |  |  |
| Eyes | 0.58 (0.41) ^a, b^ | 0.55 (0.51) ^a^ | 1.09 (0.66) ^b^ |
| Nose | 0.45 (0.29) ^a, b^ | 1.01 (0.34) ^a^ | 0.92 (0.46) ^b^ |
| Mouth | 0.73 (0.40) | 0.87 (0.41) | 0.88 (0.47) |
| Rest of Face | 0.85 (0.72) | 0.68 (0.46) | 0.81 (0.34) |
| Face | 0.72 (0.41) ^a, b^ | 0.67 (0.26) ^a^ | 0.93 (0.35) ^b^ |
| Background | 0.69 (0.37) ^a, b^ | 0.81 (0.26) ^a,^ | 0.41 (0.15) ^b^ |
| **Number of Fix. (N)** |  |  |  |
| Eyes | 228.10 (255.08) ^a, b^ | 996.35 (372.39) ^a^ | 812.62 (420.11) ^b^ |
| Nose | 224.25 (336.95) ^b^ | 213.88 (155.05) | 326.68 (249.25) ^b^ |
| Mouth | 196.00 (239.17) | 91.18 (73.84) | 223.59 (219.71) |
| Rest of Face | 332.95 (271.73) | 242.88 (206.78) | 290.92 (192.82) |
| Face | 981.30 (711.29) ^a, b^ | 1544.29 (418.78) ^a^ | 1653.81 (454.95) ^b^ |
| Background | 238.25 (277.92) ^b^ | 98.94 (75.90) | 75.41 (49.04) ^b^ |

*Note.* Mean (*M*) and standard deviation (*SD*), Autism group was split according to lower confidence interval boundary. For the group comparisons, *F*-tests for independent samples were calculated. All group comparisons are Bonferroni corrected. ^a^: **Autism** low and **Autism** high significantly differed, all *Fs*(1, 37) ≥ 10.06, *ps* ≤ .009, *η*²s ≥ .223; ^b^: **Autism** low group and Neurotypical group significantly differed, all *Fs*(1, 56) ≥ 11.82, *ps* ≤ .003 , *η*²s ≥ .182; ^c^: **Autism** high group and Neurotypical group significantly differed, all *Fs*(1, 55) ≤ 0.19, *ps* ≥ .667, *η*²s ≤ .009.

*Table S20.*

Gaze behavior parameters for Autism low, Autism high, and Neurotypical group.

|  | Autism low | Autism high | | Neurotypical |
| --- | --- | --- | --- | --- |
|  | *M (SD)* | *M (SD)* | *M (SD)* | |
| **Mutual Face Gaze** |  |  |  | |
| Total duration (%) | 12.18 (8.88) ^a,^ ^b^ | 23.58 (7.93) ^a^ | 17.42 (5.47) ^b^ | |
| Mean duration (Sec.) | 1.76 (1.65) ^a,^ ^b^ | 2.81 (2.19) ^a^ | 3.03 (1.91) ^b^ | |
| **Mutual Eye Gaze** |  |  |  | |
| Total duration (%) | 8.53 (6.62) ^a, b^ | 16.54 (5.55) ^a^ | 24.85 (7.51) ^b^ | |
| Mean duration (Sec.) | 1.64 (1.34) ^b^ | 3.17 (2.81) | 3.11 (2.27) ^b^ | |
| **Initiations** |  |  |  | |
| Total number (%) | 44.39 (18.62) ^b^ | 49.04 (18.52) ^c^ | 61.99 (10.45) ^b, c^ | |
| Mean duration (Sec.) | 2.78 (0.88) ^b^ | 3.22 (0.79) | 3.92 (1.83) ^b^ | |
| **Break ups** |  |  |  | |
| Total number (%) | 64.60 (15.01) ^b^ | 51.93 (15.80) | 41.09 (14.93) ^b^ | |

*Note.* Mean (*M*) and standard deviation (*SD*), **Autism** group was split according to lower confidence interval boundary.For the group comparisons, *F*-tests for independent samples were calculated. All group comparisons are Bonferroni corrected. ^a^: **Autism** low and **Autism** high significantly differed, all *Fs*(1, 37) ≥ 17.06, *ps* ≤ .003, *η*²s ≥ .329. ^b^: **Autism** low group and Neurotypical group significantly differed, all *Fs*(1, 56) ≥ 5.31, *ps* ≤ .025, *η*²s ≥ .091; ^c^: **Autism** high group and Neurotypical group significantly differed, all *Fs*(1, 55) ≤ 0.35, *ps* ≥ .559, *η*²s ≤ .018.

*Table S21.*

Demographic, trait, and state parameters for both autism and neurotypical groups.

|  | **Autism low** | **Autism high** | **Neurotypical** |
| --- | --- | --- | --- |
|  | *M (SD)* | *M (SD)* | *M (SD)* |
| Age | 33.61 (12.64) | 32.22 (9.60) | 33.35 (10.97) |
|  | |  |  |
| ADOS | 11.00 (2.29) ^b^ | 9.82 (2.40) ^c^ | 0.70 (1.61) ^b, c^ |
| *Communication* | 4.15 (1.35) ^b^ | 3.59 (1.41) ^c^ | 0.32 (0.78) ^b, c^ |
| *Social Interaction* | 6.51 (2.21) ^b^ | 6.53 (2.19) ^c^ | 0.38 (0.95) ^b, c^ |
| WST | 105.85 (4.88) | 106.65 (7.68) | 107.68 (5.78) |
| CFT | 115.30 (15.86) | 115.12 (16.26) | 115.16 (13.59) |
|  | |  |  |
| AQ | 37.50 (7.38) ^b^ | 32.76 (7.00) ^c^ | 14.95 (5.73) ^b, c^ |
| BFNE | 38.60 (12.27) ^b^ | 35.94 (11.73) ^c^ | 25.19 (10.28) ^b, c^ |
| SIAS | 36.50 (13.13) ^b^ | 32.76 (11.66) ^c^ | 13.87 (7.28) ^b, c^ |
| GARS | 35.05 (16.39) ^b^ | 30.71 (18.01) ^c^ | 11.54 (10.13) ^b, c^ |
| *Fear* | 14.25 (9.16) ^b^ | 13.41 (9.70) ^c^ | 5.27 (5.21) ^b, c^ |
| *Avoidance* | 20.80 (10.16) ^b^ | 17.29 (9.47) ^c^ | 6.27 (5.40) ^b, c^ |
| IRI | 56.43 (7.42) ^b^ | 58.05 (9.49) ^c^ | 52.89 (11.20) ^b, c^ |
| MINI SCL | 12.64 (3.85) ^b^ | 10.83 (2.54) ^c^ | 8.49 (0.87) ^b, c^ |

*Note.* Autism group was split according to lower confidence interval boundary. Mean (*M*) and standard deviation (*SD*), IQ (crystalline): Culture fair Test (CFT), IQ (verbal): Verbal Intelligence Test (WST), ADOS: Autism Diagnostic Observation Schedule-2 with subscales, AQ: Autism-Spectrum-Quotient. BFNE: Brief Fear of Negative Evaluation, SIAS: Social Interaction Anxiety Scale, GARS: Gaze Anxiety Rating Scale, IRI: Interpersonal Reactivity Index, MINI SCL: Mini-Symptom Check List^.^ For the group comparisons, *F*-tests for independent samples were calculated. All group comparisons are Bonferroni corrected. ^a^: **Autism** low and **Autism** high significantly differed, all *Fs*(1, 37) ≤ 2.05, *ps* ≥ .161, *η*²s ≤ .055; ^b^: **Autism** low group and Neurotypical group significantly differed, all *Fs*(1, 56) ≥ 17.82, *ps* ≤ .003, *η*²s ≥ .252; ^c^: **Autism** high group and Neurotypical group significantly differed, all *Fs*(1, 55) ≥ 15.71, *ps* ≤ .013, *η*²s ≥ 225.

*Table S22.*

Subjective ratings for groups Autism low, Autism high and Neurotypical group.

|  | **Autism low** | **Autism high** | **Neurotypical** |
| --- | --- | --- | --- |
|  | *M (SD)* | *M (SD)* | *M (SD)* |
| **pre** |  |  |  |
| Shame | 19.73 (22.61) ^b^ | 15.25 (21.31) | 7.49 (9.90) ^b^ |
| Fear | 24.10 (24.95) | 21.06 (21.59) ^c^ | 13.73 (19.85) ^c^ |
| Happy | 60.60 (26.68) ^b^ | 60.88 (23.47) ^c^ | 74.62 (22.89) ^b, c^ |
| Leave | 25.05 (30.00) | 22.71 (23.75) | 14.76 (17.01) |
| Trustee | 38.40.72 (34.01) | 33.02 (35.73) | 30.22 (26.14) |
| Stress | 39.55 (29.92) ^b^ | 32.29 (24.17) ^c^ | 23.19 (20.20) ^b, c^ |
| **post** |  |  |  |
| Shame | 17.05 (25.86) ^a, b^ | 8.24 (12.39) ^a^ | 6.43 (9.30) ^b^ |
| Fear | 20.25 (25.70) ^b^ | 15.12 (19.70) | 5.68 (7.25) |
| Happy | 68.20 (23.06) ^b^ | 69.82 (17.21) ^c^ | 82.65 (15.61) ^b, c^ |
| Leave | 20.25 (19.70) ^b^ | 15.12 (19.68) | 9.57 (14.30) |
| Trustee | 19.50 (20.72) | 22.29 (27.02) ^c^ | 14.92 (19.24) ^b, c^ |
| Stress | 25.85 (26.41) ^b^ | 17.94 (15.47) | 11.81 (14.57) ^b^ |
|  |  |  |  |
| Sympathy | 68.45 (14.72) | 75.00 (12.35) | 76.87 (15.78) |
| Authenticity | 49.80 (28.34) | 47.29 (28.17) | 56.24 (25.92) |
| Attractiveness | 46.05 (23.37) | 42.41 (24.83) | 39.32 (25.04) |
| Liking | 61.55 (16.85) | 64.18 (12.51) | 67.51 (14.59) |
| Distraction | 32.21 (28.73) | 22.16 (21.50) | 19.43 (19.16) |
| Enjoyment of int. | 55.95 (23.30) ^b^ | 69.32 (14.32) | 72.19 (13.91) ^b^ |
| Enjoyment of role | 51.20 (25.15) ^b^ | 61.59 (22.18) | 67.19 (17.27) ^b^ |
| Laughing | 39.65 (25.88) | 43.47 (19.52) | 46.70 (22.39) |
| Fun | 57.90 (25.62) ^b^ | 67.88 (10.57) | 70.86 (14.21) ^b^ |
| Revealed details (other) | 65.75 (17.45) | 60.82 (18.61) | 77.22 (14.06) |
| Revealed details | 67.83 (21.53) | 72.94 (17.41) | 62.97 (16.41) |
| Openness (other) | 74.80 (18.91) | 75.59 (15.48) | 78.95 (16.39) |
| Openness (self) | 77.95 (21.17) | 84.24 (23.77) | 85.41 (17.08) |
| IOS (actual) | 3.10 (2.08) | 2.82 (1.19) | 3.16 (1.32) |
| IOS (desired) | 2.85 (2.08) | 2.65 (1.32) | 3.00 (1.37) |

*Note.* Autism group was split according to lower confidence interval boundary. Mean (*M*) and standard deviation (*SD*). For the group comparisons, *F*-tests for independent samples were calculated. All group comparisons are Bonferroni corrected. ^a^: **Autism** low and **Autism** high significantly differed, all *Fs*(1, 37) ≤ 1.89, *ps* ≥ .178, *η*²s ≤ .051; ^b^: **Autism** low group and Neurotypical group significantly differed, all *Fs*(1, 56) ≥ 5.95 *ps* ≤ .048, *η*²s ≥ .101; ^c^: **Autism** high group and Neurotypical group significantly differed, all *Fs*(1, 55) ≥ 6.18, *ps* ≤ .048, *η*²s ≥ .103.
